# Association Between Admission Blood Pressure and In-hospital Mortality and Long-term Mortality of Patients With ST-elevation Myocardial Infarction Undergoing Percutaneous Coronary Intervention: A China Acute Myocardial Infarction Registry Study

**DOI:** 10.31083/RCM33512

**Published:** 2025-08-30

**Authors:** ZhiFeng Song, Chilie Danzeng, Yu Jiang, JinGang Yang, WeiXian Yang, HaiYan Qian, YueJin Yang

**Affiliations:** ^1^Center for Coronary Heart Disease, Department of Cardiology, Fuwai Hospital, National Center for Cardiovascular Diseases of China, State Key Laboratory of Cardiovascular Disease, Chinese Academy of Medical Sciences and Peking Union Medical College, 100037 Beijing, China; ^2^Medical Research and Biometrics Center, National Clinical Research Center for Cardiovascular Diseases, Fuwai Hospital, National Center for Cardiovascular Diseases, Peking Union Medical College and Chinese Academy of Medical Sciences, 100037 Beijing, China; ^3^Center for Coronary Heart Disease, Department of Cardiology, Beijing Anzhen Hospital, Capital Medical University, 100029 Beijing, China

**Keywords:** ST-segment elevation myocardial infarction, blood pressure, patient admission, prognosis, percutaneous coronary intervention

## Abstract

**Background:**

Globally, acute myocardial infarction (AMI) is among the primary causes of mortality. The ideal approach for blood pressure (BP) management for patients experiencing ST-segment elevation myocardial infarction (STEMI) who receive percutaneous coronary intervention (PCI) remains a topic of ongoing debate. Current guidelines on BP management lack specific recommendations for STEMI patients undergoing PCI, resulting in substantial individual variability and uncertainties in clinical treatment strategies. This research seeks to determine the ideal BP levels linked to the lowest risk of in-hospital mortality and long-term adverse endpoints in STEMI patients receiving PCI.

**Methods:**

This retrospective study analyzed data from the China Acute Myocardial Infarction (CAMI) Registry, enrolling 10,482 STEMI patients undergoing PCI at 108 Chinese hospitals from January 2013 to September 2014. The primary outcome was in-hospital mortality. Secondary outcomes included 2-year all-cause mortality, severe bleeding, and major adverse cardiac and cerebrovascular events (MACCEs), defined as a combination of all-cause mortality, myocardial infarction (MI), or stroke. The analysis of the relationship between admission systolic blood pressure (SBP)/diastolic blood pressure (DBP) and the primary and secondary outcomes as continuous and categorical variables was conducted using restricted cubic spline (RCS) analysis and Cox regression models.

**Results:**

RCS analysis revealed that a J-shaped association existed between admission SBP/DBP and the risk of the primary outcome, with significant nonlinearity (both *p* < 0.001). Both lower and higher SBP/DBP levels were linked to an elevated risk of in-hospital mortality. The ideal SBP/DBP levels to minimize the in-hospital mortality risk were 157/94 mmHg. Compared to the reference SBP/DBP group (120–129/70–79 mmHg), lower admission SBP (<109 mmHg) or DBP (60–69 mmHg) significantly elevated the risk of the primary outcome. The adjusted hazard ratio (HR) for SBP levels of 100–109 mmHg and <100 mmHg was 1.08 (95% confidence interval (CI): 1.00–1.17; *p* = 0.0395 and *p* = 0.043, respectively), and for DBP of 60–69 mmHg, the adjusted HR was 1.07 (95% CI: 1.01–1.14, *p* = 0.0305). Similarly, the J-shaped curve was also noted between SBP/DBP and secondary outcomes, such as all-cause mortality, severe bleeding and MACCEs. However, no significant non–linear relationship was observed between SBP/DBP and recurrent MI at 2-year follow-up.

**Conclusions:**

Among STEMI patients undergoing PCI, a J-curve relationship in in-hospital mortality was observed with a nadir at 157/94 mmHg. Similar J-shaped trends were also observed for secondary outcomes including all-cause mortality, severe bleeding and MACCEs. However, no significant nonlinear correlation was found between admission BP and recurrent MI within 2 years.

**Clinical Trial Registration:**

NCT01874691, https://www.clinicaltrials.gov/study/NCT01874691?term=NCT01874691&rank=1.

## 1. Introduction

Blood pressure (BP) is a significant risk factor implicated in the progression of atherosclerosis and the formation of vulnerable plaques, contributing to increased mortality in acute coronary syndrome (ACS) patients [1, 2]. Various BP parameters are validated as essential prognostic and therapeutic markers in managing cardiovascular diseases across different clinical settings. Guidelines are recommended for rigorous BP control in individuals with hypertension to minimize the morbidity and mortality of cardiovascular outcomes [3, 4]. Evidence from randomized controlled trials confirmed that reducing BP in those with hypertension decreased the risk of cardiovascular events in the future [5, 6].

Previous research suggests that while elevated BP is linked to a higher mortality risk following ACS, lower BP does not consistently demonstrate the same association. Nevertheless, achieving optimal BP control in patients after ACS remains essential for reducing subsequent cardiovascular events [7]. The SPRINT trial showed that an intensive treatment strategy aiming for a systolic blood pressure (SBP) <120 mmHg in high-risk patients significantly decreased cardiovascular mortality, thereby highlighting the benefits of aggressive BP management [8]. However, evidence indicates that universal BP management may not be appropriate for all patient populations. For example, elderly patients with an SBP below 125 mmHg had nearly a twofold risk of cardiovascular death within one year compared to those with an SBP above 125 mmHg [9]. Similarly, data from the Chinese ST-segment elevation myocardial infarction (STEMI) PPCI Registry indicated that although STEMI patients undergoing percutaneous coronary intervention (PCI) with SBP <120 mmHg experienced higher spontaneous reperfusion, the lowest all-cause mortality was observed among those with an SBP ranging from 121–150 mmHg [10]. These suggest that the correlation between BP and outcomes in acute cardiovascular settings appears complicated, as studies show inconsistent findings regarding BP levels and the risk of mortality. These studies indicated that a U- or J-shaped relationship between SBP/diastolic blood pressure (DBP) and adverse outcomes [11, 12, 13, 14, 15], suggesting that both lower BP and higher BP levels increase the risk of adverse cardiovascular outcomes. The therapeutic benefit of lowering BP may be reversed if BP is reduced below a certain threshold [16, 17].

These conflicting results highlight persistent uncertainties regarding the association between BP levels and the cardiovascular mortality risk of ACS patients. Also, the precise BP levels that correlate with the minimal mortality risk remain poorly defined. Currently, evidence on the prognostic impact of admission SBP/DBP on in-hospital mortality and long-term outcomes in STEMI patients undergoing PCI is limited. Utilizing the data sourced from the large-scale China Acute Myocardial Infarction (CAMI) registry, our study represents the first investigation to employ restricted cubic spline (RCS) analysis to systematically determine the association between levels of admission SBP/DBP and the risk of both in-hospital mortality and long-term endpoints in a large Chinese STEMI cohort. Based on prior evidence, we hypothesize that a J-shaped relationship between admission SBP/DBP and in-hospital mortality, aiming to identify the optimal admission BP levels linked to the lowest mortality risk in STEMI patients undergoing PCI.

## 2. Study Design

This CAMI study is a large-scale, prospective, multicenter observational study in China, aimed at gathering real-world clinical data from acute myocardial infarction (AMI) patients (NCT01874691) [18]. This project was approved by the central institutional review board at Fuwai Hospital and by the ethics committees at each participating institution. Written informed consent was obtained from every enrolled participant. The Data Monitoring Committee was set to supervise data and ensure its quality. The registry includes 108 hospitals across 31 provinces and municipalities in mainland China, with Hong Kong and Macau not included. Eligible patients primarily diagnosed with AMI were consecutively recruited in the registry from January 2013 to September 2014. The AMI diagnosis was rigorously defined based on the third Universal Definition for Myocardial Infarction, including types 1, 2, 3, 4b, and 4c [19]. Type 4a and type 5 AMI were not included in this registry.

Data collected encompassed patient demographics, clinical risk factors, physical clinical examination findings, discharge medications and laboratory results [18]. The discharge medications specifically encompassed β-blockers, dual antiplatelet therapy (aspirin/clopidogrel), statins, and angiotensin-converting enzyme inhibitors (ACEis)/angiotensin receptor blockers (ARBs). The recorded laboratory parameters included total cholesterol (TC), low-density lipoprotein cholesterol (LDL-C), triglycerides (TG), high-density lipoprotein cholesterol (HDL-C), N-terminal pro-B-type natriuretic peptide (NT-pro BNP), white blood cell count (WBC), high-sensitivity C-reactive protein (Hs-CRP), and Troponin I (TnI). Extensive information on managing data and ensuring its quality has been previously detailed in the methodological sections of an earlier publication on the CAMI registry [18].

### 2.1 Study Population and Definitions

**Fig. 1.**
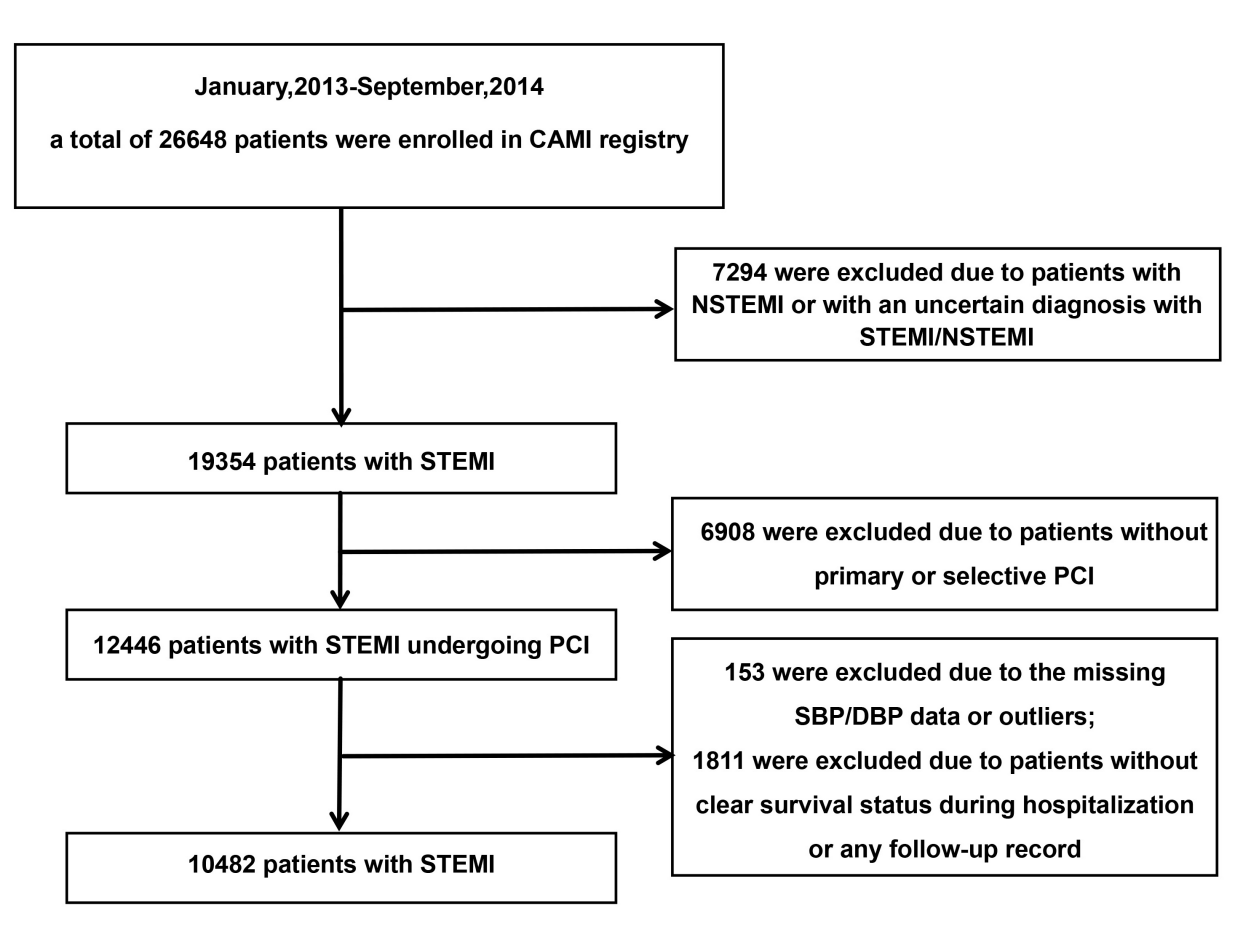
**The flowchart of criteria for including and excluding participants**. CAMI, China Acute Myocardial Infarction; NSTEMI, Non-ST segment elevation myocardial infarction; STEMI, ST-segment elevation myocardial infarction; PCI, percutaneous coronary intervention; SBP, systolic blood pressure; DBP, diastolic blood pressure.

The CAMI registry recorded 26,648 AMI patients from January 2013 to September 2014. The inclusion criteria encompassed those diagnosed with AMI. The excluded criteria included: those with non-STsegment elevation myocardial infarction (NSTEMI) or an uncertain diagnosis with STEMI/NSTEMI (n = 7294); patients without primary or selective PCI (n = 6908); missing or outlier SBP/DBP values (n = 153); or those lacking clear survival status during hospitalization or any follow-up data (n = 1811). Ultimately, a total of 10,482 STEMI patients undergoing PCI were included in subsequent analysis (Fig. 1).

Diabetes mellitus was defined as a documented history of the condition, prior hypoglycemic treatment or an admission hemoglobin A1c (HbA1c) level of 6.5% or higher [20]. Upon first admission to the Cardiology Department of FuWai Hospital, patients were instructed to rest quietly for at least five minutes before BP measurement. BP was measured by professional cardiologists with a validated electronic BP monitor. During the measurement, the upper arms of the patients were placed at the same height as the heart. Measurements were taken from both arms, and the higher value was recorded. Hyperlipidemia was characterized by plasma triglyceride levels of at least 200 mg/dL, total cholesterol levels of at least 240 mg/dL, or a recorded history of lipid-lowering medication use prior to admission [21].

### 2.2 Primary and Secondary Outcomes

During the 2-year follow-up after inclusion in the CAMI registry, the primary outcome was in-hospital mortality. The secondary outcomes included 2-year heart failure, 2-year all-cause mortality, 2-year severe bleeding, and 2-year MACCEs. MACCEs were defined as a combination of all-cause mortality, recurrent MI, and ischemic stroke. Rehospitalization or doctor visits for heart failure (HF) were considered indicative of new or worsening HF, diagnosed by clinical symptoms such as cardiac dyspnea and pink frothy sputum and was supported by laboratory tests. The identification of severe bleeding events followed the Bleeding Academic Research Consortium (BARC) criteria, with BARC types 0, 1, and 2 excluded [22].

### 2.3 Statistical Analysis

Variables that are continuous and normally distributed are represented as mean ± standard deviation and analyzed by Student *t*-test. The normal distribution was verified using Kolmogorow-Smironov test. Continuous variables with non-normal distribution were compared using Mann-Whitney U-test, and the data are presented as median (interquartile range, IQR). Categorical variables were represented by frequencies and percentages, with comparisons performed using chi-square or Fisher’s exact test. To evaluate the impact of admission SBP/DBP on in-hospital mortality and long-term outcomes, including all-cause mortality, severe bleeding, and MACCEs during the 2-year follow-up, the multivariate Cox regression models were constructed. The models incorporated covariates such as age, body mass index (BMI), gender, admitted heart rate, hyperlipidemia, diabetes mellitus, smoking and alcohol consumption history, heart failure (HF), myocardial infarction (MI), stroke, creatinine clearance rate and left ventricular ejection fraction (LVEF). Moreover, logistic regression analysis was applied to determine the relationship between levels of admission SBP/DBP and heart failure at 24 months with adjustments for the aforementioned variables.

Given the potential non-linear relationship between admission SBP/DBP and outcomes, RCS analysis with three knots was conducted. The knots of the SBP group were determined as 98 mmHg (10th), 125 mmHg (50th), 160 mmHg (90th), while the DBP group knots were determined as 60 mmHg (10th), 79 mmHg (50th), 100 mmHg (90th) by using a percentile-based method, respectively. This spline analysis evaluates the continuous associations between admission SBP/DBP and all outcomes and identified the levels of admission SBP/DBP with the lowest risk of outcomes. Interaction testing using Cox regression models was conducted for subgroup analyses categorized by age (<65 years, ≥65 years), gender (male or female) and diabetes. These subgroup analyses utilized identical knot placements as the primary analyses to facilitate direct comparison. Additionally, the SBP/DBP of the patients were first categorized into seven groups, ranging from 100–150 mmHg/60–100 mmHg with each spanning an interval of 10 mmHg. The admission SBP of 120–129 mmHg and DBP of 70–79 mmHg were selected as the reference groups following findings from previous studies [17, 23].

For Cox proportional hazards regression models and logistic regression models, missing qualitative indicators are imputed by the most frequent category, whereas quantitative indicators are filled with the mean value. **Supplementary Table 1 and Table 2** presents a summary of missing data for all covariates and compares baseline characteristics between participants with and without missing data to evaluate potential systematic differences. Statistical analyses utilized SAS software (version 9.4 for Windows, SAS Institute Inc., Cary, NC, USA). Statistical significance in a 2-tailed test was determined by a *p*-value of less than 0.05.

## 3. Results

### 3.1 Baseline Characteristics

After excluding 16,166 patients based on predefined criteria, the current study involved 10,482 eligible STEMI patients. The clinical and demographic characteristics of the cohort are detailed in Tables 1,2. The participants had a mean age of 59.84 ± 11.78 years, with males comprising 80.6% (n = 8450) of the study population. The most prevalent comorbidity was diabetes (18.5%), followed by dyslipidemia (8.6%).

**Table 1.** **Demographic and baseline characteristics of the patients by mean systolic blood pressure categories**.

	Systolic blood pressure groups								*p*-value
	Total	<100 mmHg	100–109 mmHg	110–119 mmHg	120–129 mmHg	130–139 mmHg	140–149 mmHg	≥150 mmHg	
N (total = 10,482)	10,482	1133	1139	1644	1808	1632	1223	1903	–
Age (years)	60.02 (51.06–68.25)	61.20 (53.59–70.12)	59.94 (50.74–68.00)	59.38 (50.32–67.33)	59.42 (50.33–67.79)	59.87 (51.00–67.43)	59.76 (50.81–67.44)	61.13 (51.97–69.66)	<0.0001
Male sex	8450 (80.6%)	904 (79.8%)	949 (83.3%)	1370 (83.3%)	1475 (81.6%)	1321 (80.9%)	980 (80.1%)	1451 (76.2%)	<0.0001
BMI (kg/m^2^)	24.22 (22.49–26.04)	23.88 (22.03–25.72)	23.94 (22.09–25.71)	24.21 (22.49–25.95)	24.22 (22.49–25.95)	24.22 (22.58–26.12)	24.38 (22.68–26.11)	24.49 (22.49–26.53)	<0.0001
Baseline SBP (mmHg)	125.00 (110.00–141.00)	90.00 (85.00–96.00)	104.00 (100.00–106.00)	113.00 (110.00–116.00)	122.00 (120.00–126.00)	132.00 (130.00–136.00)	142.00 (140.00–145.00)	160.00 (154.00–171.00)	<0.0001
Baseline DBP (mmHg)	79.00 (70.00–90.00)	60.00 (52.00–63.00)	67.00 (61.00–71.00)	71.00 (68.00–78.00)	78.00 (70.00–82.00)	80.00 (76.50–90.00)	90.00 (80.00–95.00)	98.00 (89.00–106.00)	<0.0001
Heartrate (b.p.m.)	75.00 (65.00–86.00)	66.00 (52.00–63.00)	72.00 (62.00–83.00)	74.00 (65.00–84.00)	76.00 (66.00–86.00)	76.00 (67.00–86.00)	78.00 (68.00–88.00)	79.00 (70.00–90.00)	<0.0001
LVEF (%)	55.00 (48.00–60.00)	54.00 (46.00–60.00)	54.00 (47.00–60.00)	55.00 (47.48–60.00)	54.00 (47.00–60.00)	55.00 (48.00–60.00)	55.00 (48.00–60.00)	56.00 (49.00–61.22)	<0.0001
Killip III/IV	547 (5.2%)	218 (19.3%)	53 (4.7%)	81 (4.9%)	51 (2.8%)	47 (2.9%)	31 (2.5%)	66 (3.5%)	<0.0001
Mean glucose	7.00 (5.72–9.10)	7.30 (5.87–9.80)	6.77 (5.70–8.70)	6.80 (5.60–8.73)	6.87 (5.54–8.91)	7.01 (5.80–9.00)	7.20 (5.79–9.50)	7.17 (5.83–9.41)	0.2415
LDL-C (mmol/L)	2.78 (2.23–3.37)	2.63 (2.10–3.20)	2.68 (2.19–3.27)	2.74 (2.20–3.35)	2.79 (2.23–3.40)	2.84 (2.26–3.43)	2.82 (2.30–3.40)	2.87 (2.32–3.47)	0.0122
HDL-C (mmol/L)	1.00 (0.90–1.20)	1.00 (0.80–1.20)	1.00 (0.80–1.20)	1.00 (0.80–1.20)	1.00 (0.80–1.20)	1.00 (0.90–1.30)	1.00 (0.90–1.20)	1.10 (0.90–1.30)	0.5998
Total Cholesterol	4.53 (3.85–5.25)	4.28 (3.67–4.98)	4.41 (3.71–5.09)	4.49 (3.81–5.20)	4.51 (3.82–5.24)	4.67 (3.96–5.37)	4.61 (3.99–5.33)	4.65 (3.95–5.40)	<0.0001
Triglycerides	1.44 (1.01–2.10)	1.30 (0.88–1.89)	1.42 (0.99–2.03)	1.40 (1.02–2.05)	1.45 (1.02–2.05)	1.46 (1.04–2.18)	1.51 (1.07–2.22)	1.49 (1.03–2.23)	0.1029
Hs-CRP (mg/L)	6.35 (2.46–14.60)	7.15 (2.91–22.30)	8.20 (2.60–16.49)	6.52 (2.80–14.32)	6.24 (2.10–14.33)	5.75 (2.35–13.39)	6.36 (2.27–14.84)	6.00 (2.52–13.89)	<0.0001
NT-proBNP (ng/ml)	457.8 (125.00–1420.0)	674.00 (191.00–2066.00)	488.00 (125.00–1534.00)	490.50 (127.00–1523.00)	405.00 (122.00–1344.00)	436.00 (114.00–1407.00)	374.80 (125.00–1078.00)	439.00 (118.00–1342.00)	<0.0001
WBC (×10^9^/L)	10.10 (8.10–12.57)	10.90 (8.50–13.70)	10.21 (8.10–12.78)	10.31 (8.23–12.81)	10.00 (8.01–12.49)	9.93 (8.09–12.30)	10.00 (7.94–12.30)	9.74 (7.96–12.05)	<0.0001
PLT (×10^9^/L)	207.00 (172.00–246.00)	199.00 (166.00–244.00)	201.00 (167.00–239.00)	206.00 (170.00–244.00)	206.00 (171.00–246.00)	211.00 (177.00–251.00)	209.00 (175.00–251.50)	211.00 (174.00–248.00)	0.0014
Serum creatinine (mmol/L)	73.60 (61.90–87.90)	79.90 (66.00–100.90)	74.60 (63.25–88.90)	74.00 (62.10–86.80)	72.40 (60.00–86.00)	73.00 (61.00–86.00)	71.50 (60.80–85.00)	72.00 (61.00–86.50)	<0.0001
Previous MI	520 (5.2%)	80 (7.5%)	71 (6.5%)	82 (5.2%)	78 (4.5%)	68 (4.4%)	57 (4.9%)	84 (4.7%)	0.0051
TnI (ng/mL)	25.07 (6.37–50.00)	32.00 (11.80–50.00)	28.27 (9.96–50.00)	26.85 (6.50–50.00)	20.47 (6.62–46.30)	26.75 (6.17–50.00)	20.48 (4.89–50.00)	22.17 (4.34–50)	0.4106
History of heart failure	78 (0.8%)	14 (1.3%)	8 (0.7%)	11 (0.7%)	14 (0.8%)	12 (0.8%)	11 (0.9%)	8 (0.4%)	0.348
Admission heart failure	1151 (11.1%)	231 (20.6%)	125 (11.0%)	183 (11.2%)	168 (9.4%)	136 (8.4%)	95 (7.8%)	214 (11.3%)	<0.0001
Peripheral vascular disease	43 (0.4%)	3 (0.3%)	3 (0.3%)	12 (0.8%)	7 (0.4%)	5 (0.3%)	7 (0.6%)	6 (0.3%)	0.3965
Hyperlipidemia	807 (8.6%)	73 (7.1%)	91 (8.9%)	134 (8.9%)	135 (8.3%)	133 (9%)	101 (9.2%)	140 (8.5%)	0.6414
Diabetes	1880 (18.5%)	179 (16.2%)	168 (15.3%)	280 (17.4%)	302 (17.1%)	305 (19.4%)	269 (22.7%)	377 (20.7%)	<0.0001
Prior stroke	793 (7.8%)	66 (6.0%)	62 (5.6%)	118 (7.4%)	137 (7.8%)	123 (7.8%)	99 (8.4%)	188 (10.4%)	0.0001
COPD	139 (1.4%)	20 (1.8%)	17 (1.5%)	20 (1.3%)	26 (1.5%)	20 (1.3%)	14 (1.2%)	22 (1.2%)	0.8268
Haemoglobin (g/dL)	139.84 ± 19.27	134.43 ± 20.48	136.55 ± 19.38	138.73 ± 18.72	139.61 ± 18.48	141.27 ± 17.67	142.53 ± 18.39	143.24 ± 20.41	<0.0001
ACEi/ARB	581 (6.2%)	49 (4.8%)	59 (5.7%)	80 (5.4%)	90 (5.5%)	95 (6.5%)	70 (6.5%)	138 (8.4%)	0.0026
Beta-blocker	443 (4.7%)	46 (4.5%)	54 (5.1%)	66 (4.4%)	77 (4.7%)	65 (4.5%)	52 (4.8%)	83 (5.0%)	0.9667
Aspirin	826 (8.6%)	88 (8.5%)	94 (8.9%)	132 (8.7%)	151 (9%)	114 (7.7%)	100 (8.9%)	147 (8.6%)	0.8982
Clopidogrel	340 (3.5%)	38 (3.7%)	45 (4.2%)	51 (3.3%)	63 (3.7%)	38 (2.6%)	46 (4.1%)	59 (3.5%)	0.2806
Diuretics	85 (0.9%)	14 (1.4%)	8 (0.8%)	19 (1.3%)	11 (0.7%)	12 (0.8%)	9 (0.8%)	12 (0.7%)	0.3740
CCB	687 (7.3%)	63 (6.2%)	42 (4.0%)	91 (6.1%)	102 (6.2%)	115 (7.9%)	99 (9.1%)	175 (10.5%)	<0.0001

Continuous variables are medians with 25th and 75th percentiles. Abbreviations: BMI, body mass index; SBP, systolic blood pressure; DBP, diastolic blood pressure; LVEF, left ventricular ejection fraction; LDL-C, low-density lipoprotein cholesterol; HDL-C, high-density lipoprotein cholesterol; Hs-CRP, high-sensitivity C-reactive protein; NT-proBNP, N-terminal pro-brain natriuretic peptide; WBC, white blood cell; PLT, platelet; MI, myocardial infarction; TnI, Troponin I/T; COPD, chronic obstructive pulmonary disease; ACEi/ARB, angiotensin-converting enzyme inhibitor/angiotensin receptor blocker; CCB, calcium-channel blocker.

**Table 2.** **Demographic and baseline characteristics of the patients by mean diastolic blood pressure categories**.

	Diastolic blood pressure groups							*p*-value
	<60 mmHg	60–69 mmHg	70–79 mmHg	80–89 mmHg	90–99 mmHg	100–109 mmHg	≥110 mmHg	
N (total = 10,482)	807	1806	2642	2588	1469	759	411	–
Age (years)	63.79 (55.33–72.58)	60.95 (51.58–69.63)	61.00 (52.42–68.63)	60.00 (51.09–67.85)	58.87 (50.35–66.71)	56.61 (48.59–64.03)	54.92 (47.13–63.68)	<0.0001
Male sex	621 (77.0%)	1462 (81.0%)	2113 (80.0%)	2078 (80.3%)	1198 (81.6%)	628 (82.7%)	350 (85.2%)	0.0113
BMI (kg/m^2^)	23.88 (22.04–25.67)	23.88 (22.06–25.78)	24.22 (22.49–25.95)	24.22 (22.53–26.03)	24.34 (22.76–26.17)	24.68 (22.69–26.80)	24.92 (22.86–27.08)	<0.0001
Baseline SBP (mmHg)	91.00 (81.00–102.00)	106.00 (98.00–116.00)	120.00 (110.00–130.00)	130.00 (120.00–140.00)	142.00 (134.00–155.00)	155.00 (145.00–166.00)	174.00 (163.00–189.00)	<0.0001
Baseline DBP (mmHg)	53.00 (50.00–57.00)	64.00 (60.00–67.00)	72.00 (70.00–76.00)	81.00 (80.00–85.00)	92.00 (90.00–95.00)	100.00 (100.00–105.00)	115.00 (110.00–120.00)	<0.0001
Heartrate (b.p.m.)	62.00 (51.00–77.00)	70.00 (61.00–82.00)	74.00 (65.00–84.00)	76.00 (68.00–86.00)	80.00 (70.00–90.00)	80.00 (71.00–92.00)	84.00 (74.00–95.00)	<0.0001
LVEF (%)	55.00 (48.00–60.00)	55.00 (48.00–60.00)	55.00 (47.00–60.00)	55.00 (48.00–60.00)	55.00 (48.00–60.00)	55.00 (47.61–60.00)	54.00 (48.00–60.00)	0.9185
Killip III/IV	180 (22.4%)	97 (5.4%)	112 (4.3%)	81 (3.1%)	42 (2.9%)	17 (2.2%)	18 (4.4%)	<0.0001
Mean glucose (mmol/L)	7.44 (5.96–10.00)	6.70 (5.62–8.79)	6.91 (5.69–9.00)	7.01 (5.74–9.10)	7.10 (5.80–9.06)	7.01 (5.76–9.26)	7.24 (5.80–9.65)	0.0394
LDL-C (mmol/L)	2.61 (2.09–3.20)	2.68 (2.15–3.24)	2.76 (2.21–3.34)	2.80 (2.26–3.43)	2.90 (2.33–3.49)	2.87 (2.31–3.49)	2.93 (2.44–3.54)	0.0165
HDL-C (mmol/L)	1.00 (0.80–1.20)	1.00 (0.80–1.20)	1.00 (0.80–1.20)	1.00 (0.90–1.20)	1.00 (0.90–1.20)	1.10 (0.90–1.30)	1.10 (0.90–1.30)	0.6042
Total Cholesterol (mmol/L)	4.27 (3.65–4.96)	4.43 (3.73–5.12)	4.44 (3.81–5.18)	4.58 (3.90–5.31)	4.71 (4.00–5.40)	4.70 (4.00–5.41)	4.72 (4.09–5.47)	<0.0001
Triglycerides (mmol/L)	1.28 (0.88–1.87)	1.35 (0.97–1.94)	1.42 (1.00–2.07)	1.46 (1.04–2.11)	1.56 (1.08–2.27)	1.46 (1.06–2.25)	1.66 (1.10–2.39)	0.1800
Hs-CRP (mg/L)	7.49 (3.02–19.70)	7.93 (2.94–18.20)	6.23 (2.32–14.19)	6.47 (2.45–13.98)	6.03 (2.00–14.04)	5.12 (2.39–11.96)	5.93 (2.51–14.40)	<0.0001
NT-proBNP (fmol/L)	660.75 (179.10–1797.00)	506.75 (144.00–1744.00)	487.86 (131.00–1487.00)	410.00 (104.00–1305.00)	374.66 (121.40–1197.00)	404.50 (109.00–1198.00)	337.30 (96.68–1184.00)	0.0001
WBC (×10^9^/L)	10.75 (8.34–13.89)	10.10 (8.07–12.71)	10.06 (8.10–12.40)	10.07 (8.00–12.48)	10.09 (8.27–12.40)	9.93 (8.08–12.60)	10.03 (8.18–12.00)	<0.0001
PLT (×10^9^/L)	197.00 (164.00–241.00)	204.00 (168.00–245.00)	203.50 (169.00–242.00)	209.00 (174.00–248.00)	212.00 (177.00–248.00)	214.00 (177.00–255.00)	214.00 (178.50–251.00)	<0.0001
Serum creatinine (mmol/L)	81.30 (68.00–105.52)	74.00 (62.30–88.00)	73.00 (61.40–87.48)	72.10 (60.00–86.00)	72.00 (60.80–84.00)	72.00 (61.70–84.00)	73.00 (62.00–89.00)	<0.0001
Previous MI	57 (7.5%)	99 (5.8%)	132 (5.3%)	122 (5.0%)	67 (4.8%)	27 (3.8%)	16 (4.1%)	0.0432
TnI (ng/mL)	32.00 (10.75–50.00)	27.79 (9.23–50.00)	22.64 (6.54–50.00)	22.78 (5.58–50.00)	24.72 (5.54–50.00)	22.28 (5.21–50.00)	27.31 (4.86–50.00)	0.1635
History of heart failure	7 (0.9%)	18 (1.0%)	24 (1.0%)	14 (0.6%)	9 (0.6%)	5 (0.7%)	1 (0.3%)	0.3891
Admission heart failure	166 (20.8%)	216 (12.0%)	263 (10.0%)	247 (9.6%)	147 (10.1%)	68 (9.0%)	45 (11.0%)	<0.0001
Peripheral vascular disease	4 (0.5%)	9 (0.5%)	12 (0.5%)	12 (0.5%)	3 (0.2%)	3 (0.4%)	0 (0.0%)	0.4247
Hyperlipidemia	68 (9.3%)	139 (8.5%)	205 (8.6%)	204 (8.7%)	110 (8.5%)	49 (7.5%)	32 (9.0%)	0.9445
Diabetes	149 (18.9%)	300 (17.1%)	526 (20.5%)	448 (17.9%)	256 (18.1%)	137 (18.9%)	64 (16.2%)	0.0726
Prior stroke	50 (6.4%)	132 (7.6%)	188 (7.4%)	200 (8.1%)	118 (8.3%)	72 (9.8%)	33 (8.4%)	0.2520
COPD	12 (1.5%)	26 (1.5%)	35 (1.4%)	37 (1.5%)	19 (1.3%)	4 (0.5%)	6 (1.5%)	0.4823
Haemoglobin (g/dL)	133.37 ± 20.31	135.70 ± 19.37	138.09 ± 18.24	140.62 ± 18.84	143.76 ± 18.20	146.38 ± 18.93	150.97 ± 18.98	<0.0001
ACEi/ARB	38 (5.3%)	110 (6.7%)	130 (5.4%)	144 (6.3%)	85 (6.5%)	45 (6.5%)	29 (8%)	0.3796
Beta-blocker	37 (5.1%)	80 (4.9%)	101 (4.2%)	108 (4.7%)	70 (5.3%)	29 (4.2%)	18 (4.9%)	0.7756
Aspirin	70 (9.6%)	163 (9.8%)	214 (8.8%)	194 (8.3%)	95 (7.1%)	53 (7.5%)	37 (9.9%)	0.1193
Clopidogrel	30 (4.1%)	79 (4.7%)	78 (3.2%)	75 (3.2%)	45 (3.3%)	20 (2.8%)	13 (3.5%)	0.1231
Diuretics	8 (1.1%)	15 (0.9%)	24 (1.0%)	21 (0.9%)	11 (0.8%)	3 (0.4%)	3 (0.8%)	0.8302
CCB	49 (6.8%)	92 (5.6%)	158 (6.6%)	157 (6.8%)	126 (9.6%)	65 (9.3%)	40 (11.0%)	<0.0001

Continuous variables are medians with 25th and 75th percentiles.

The mean admission SBP/DBP for the cohort was 127.26 ± 24.67 mmHg and 78.79 ± 15.75 mmHg, respectively. Across all SBP groups, the proportion of males was consistently higher. Patients with lower SBP (SBP <110 mmHg) were older and had significantly higher levels of white blood count, Hs-CRP, serum creatinine, NT-pro BNP compared to patients with a SBP >110 mmHg. They also had higher rates of heart failure and cardiac arrest upon admission (all *p* < 0.05). Conversely, they exhibited lower levels of TG, LDL-C, and LVEF, as well as a lower prevalence of comorbidities such as diabetes and stroke (all *p* < 0.05). They were less likely to receive calcium channel blockers (CCBs) and ACEis/ARBs before admission due to STEMI (Table 3). Patients with a DBP <70 mmHg presented with lower BMIs, admitted heart rate and LDL-C. They were more likely to have a history of using antiplatelet drugs, such as aspirin and clopidogrel (all *p* < 0.05) (Table 4).

**Table 3.** **Demographic and baseline characteristics of the patients by mean systolic blood pressure categories (<110 mmHg vs ≥110 mmHg)**.

	Systolic blood pressure		*p*-value
	<110 mmHg	≥110 mmHg	
N (total = 10,482)	2272	8210	–
Age (years)	60.54 (51.72–69.05)	59.94 (50.93–68.02)	<0.001
Male sex	1853 (81.6%)	6597 (80.4%)	0.1965
BMI (kg/m^2^)	23.88 (22.04–25.71)	24.22 (22.53–26.12)	<0.001
Baseline SBP (mmHg)	100 (90.00–104.00)	131 (120.00–147.00)	<0.001
Baseline DBP (mmHg)	62.00 (58.00–69.00)	80.00 (74.00–90.00)	<0.001
Heart rate (b.p.m.)	70.00 (59.00–82.00)	76.00 (67.00–87.00)	<0.001
LVEF (%)	54.00 (47.00–60.00)	55.00 (48.00–60.00)	<0.001
Killip III/IV (%)	271 (12.0%)	276 (3.4%)	<0.001
Mean glucose (mmol/L)	7.00 (5.76–9.26)	7.00 (5.71–9.09)	0.1029
LDL-C (mmol/L)	2.65 (2.14–3.23)	2.81 (2.26–3.41)	<0.001
HDL-C (mmol/L)	1.00 (0.80–1.20)	1.00 (0.90–1.20)	0.5829
Total cholesterol (mmol/L)	4.34 (3.69–5.02)	4.58 (3.90–5.31)	<0.001
Triglycerides (mmol/L)	1.36 (0.94–1.96)	1.46 (1.03–2.14)	<0.001
Hs-CRP (mg/L)	7.58 (2.85–19.40)	6.04 (2.40–13.94)	<0.001
NT-proBNP (fmol/L)	581.00 (151.91–1710.00)	426.30 (118.92–1352.00)	<0.001
WBC (×10^9^/L)	10.50 (8.30–13.20)	10.00 (8.04–12.40)	<0.001
PLT (×10^9^/L)	200.00 (167.00–241.00)	208.00 (173.00–248.00)	<0.001
Serum creatinine (mmol/L)	77.00 (65.00–94.00)	72.50 (61.00–86.00)	<0.001
Previous MI	151 (7.0%)	369 (4.7%)	<0.001
TnI (ng/mL)	30.00 (10.60–50.00)	23.25 (5.71–50.00)	0.6479
History of heart failure	22 (1.0%)	56 (0.7%)	0.1735
Admission heart failure	356 (15.8%)	796 (9.8%)	<0.001
Admission cardiogenic shock	225 (10.0%)	72 (0.9%)	<0.001
Admission cardiac arrest	40 (1.8%)	67 (0.8%)	<0.001
Peripheral vascular disease	6 (0.3%)	37 (0.5%)	0.1909
Hyperlipidemia	164 (8.0%)	643 (8.7%)	0.2858
Diabetes	347 (15.8%)	1533 (19.3%)	<0.001
Prior stroke	128 (5.8%)	665 (8.4%)	<0.001
COPD	37 (1.7%)	102 (1.3%)	0.1695
Haemoglobin (g/dL)	136.70 (125.00–148.00)	142.00 (130.00–153.00)	<0.001
ACEi/ARB	108 (5.2%)	473 (6.4%)	0.0393
Beta-blocker	100 (4.8%)	343 (4.7%)	0.7622
Aspirin	182 (8.7%)	644 (8.6%)	0.8654
Clopidogrel	83 (4.0%)	257 (3.4%)	0.2423
Diuretics	22 (1.1%)	63 (0.8%)	0.3727
CCB	105 (5.1%)	582 (7.9%)	<0.001

Continuous variables are medians with 25th and 75th percentiles.

**Table 4.** **Demographic and baseline characteristics of the patients by mean diastolic blood pressure categories (<70 mmHg vs ≥70 mmHg)**.

	Diastolic blood pressure		*p*-value
	<70 mmHg	≥70 mmHg	
N (total = 10,482)	2613	7869	-
Age (years)	61.61 (52.44–70.67)	59.51 (50.73–67.48)	<0.001
Male sex	2083 (79.7%)	6367 (80.9%)	0.1821
BMI (kg/m^2^)	23.88 (22.04–25.71)	24.22 (22.53–26.12)	<0.001
Baseline SBP (mmHg)	102.00 (93.00–113.00)	131.00 (120.00–148.00)	<0.001
Baseline DBP (mmHg)	60.00 (58.00–65.00)	81.00 (76.00–91.00)	<0.001
Heart rate (b.p.m.)	68.00 (59.00–80.00)	77.00 (68.00–88.00)	<0.001
LVEF (%)	55.00 (48.00–60.00)	55.00 (48.00–60.00)	0.9680
Killip III/IV (%)	277 (10.6%)	270 (3.4%)	<0.001
Mean glucose (mmol/L)	6.93 (5.70–9.17)	7.00 (5.73–9.10)	0.5737
LDL-C (mmol/L)	2.65 (2.13–3.23)	2.82 (2.26–3.42)	<0.001
HDL-C (mmol/L)	1.00 (0.80–1.20)	1.00 (0.90–1.20)	0.5829
Total cholesterol (mmol/L)	4.37 (3.70–5.08)	4.58 (3.91–5.30)	<0.001
Triglycerides (mmol/L)	1.34 (0.94–1.93)	1.48 (1.04–2.16)	0.3629
Hs-CRP (mg/L)	7.81 (2.96–18.61)	6.00 (2.31–13.84)	<0.001
NT-proBNP (fmol/L)	567.53 (155.00–1769.00)	413.00 (116.00–1333.00)	<0.001
WBC (×10^9^/L)	10.32 (8.10–13.00)	10.06 (8.10–12.43)	<0.001
PLT (×10^9^/L)	202.00 (167.00–244.00)	208.00 (173.00–247.00)	<0.001
Serum creatinine (mmol/L)	76.00 (64.00–92.35)	72.50 (61.00–86.00)	<0.001
Previous MI	156 (6.3%)	364 (4.9%)	<0.001
TnI (ng/mL)	29.77 (9.78–50.00)	23.00 (5.80–50.00)	0.6479
History of heart failure	25 (1.0%)	53 (0.7%)	0.1735
Admission heart failure	382 (14.7%)	770 (9.9%)	<0.001
Admission cardiogenic shock	228 (8.8%)	69 (0.9%)	<0.001
Admission cardiac arrest	37 (1.4%)	70 (0.9%)	0.0250
Peripheral vascular disease	13 (0.5%)	30 (0.4%)	0.4437
Hyperlipidemia	207 (8.8%)	600 (8.5%)	0.7359
Diabetes	449 (17.6%)	1431 (18.8%)	0.1736
Prior stroke	182 (7.2%)	611 (8.1%)	0.1619
COPD	38 (1.5%)	101 (1.3%)	0.5296
Haemoglobin (g/dL)	134.99 ± 19.69	141.45 ± 18.86	<0.001
ACEi/ARB	148 (6.3%)	433 (6.2%)	0.8275
Beta-blocker	117 (4.9%)	326 (4.6%)	0.5430
Aspirin	233 (9.7%)	593 (8.3%)	0.0287
Clopidogrel	83 (4.0%)	257 (3.4%)	0.0027
Diuretics	23 (1.0%)	62 (0.9%)	0.6633
CCB	141 (6.0%)	546 (7.7%)	0.0036

Continuous variables are medians with 25th and 75th percentiles.

### 3.2 SBP/DBP and In-hospital Mortality

**Fig. 2.**
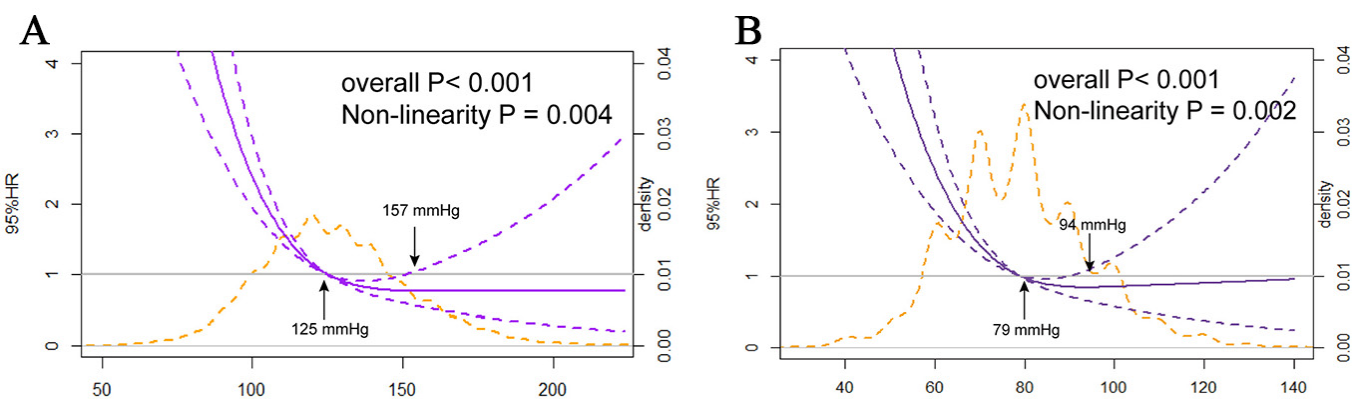
**The nonlinear J-shaped relationship between SBP/DBP levels and in-hospital mortality**. (A) Systolic blood pressure. (B) Diastolic blood pressure.

The association between admission SBP levels and the in-hospital mortality risk in STEMI patients receiving PCI followed a non-linear J-shaped trend, as assessed by restricted cubic spline analysis (*p* for nonlinearity = 0.004), with a nadir at 157 mmHg (Fig. 2).

Different SBP groups were analyzed using a Cox proportional hazards model to explore this relationship further. Among the total cohort, compared with individuals whose SBP fell within the 120–130 mmHg range, the patients with an SBP below 100 mmHg and those with an SBP ranging from 100–110 mmHg exhibited an increased risk of in-hospital mortality [HR = 1.08 (95% CI: 1.00–1.17), *p* < 0.05 for both groups] (Table 5). No significant differences in in-hospital mortality were found among patients within the other SBP categories.

**Table 5.** **Adjusted hazard ratios by mean systolic blood pressure and diastolic blood pressure categories**.

Categories	Endpoint	BP quartiles (mmHg)	Events (n/Total)	Mortality (%)	Adjusted hazard ratio (95% CI)	
					Adjusted	*p*-value
Systolic blood pressure	In-hospital mortality	<100 mmHg	41/1133	3.62	1.08 (1.00–1.17)	0.0430
		100–109 mmHg	17/1139	1.49	1.08 (1.00–1.17)	0.0395
		110–119 mmHg	22/1644	1.34	1.05 (0.98–1.12)	0.1501
		120–129 mmHg	22/1808	1.22	Reference	–
		130–139 mmHg	9/1632	0.55	1.01 (0.95–1.08)	0.6982
		140–149 mmHg	5/1223	0.41	1.02 (0.95–1.10)	0.6427
		≥150 mmHg	20/1903	1.05	1.00 (0.94–1.07)	0.9643
Diastolic blood pressure	In-hospital mortality	<60 mmHg	33/804	4.09	1.05 (0.97–1.14)	0.2042
		60–69 mmHg	23/1806	1.27	1.07 (1.01–1.14)	0.0305
		70–79 mmHg	30/2642	1.14	Reference	–
		80–89 mmHg	28/2588	1.08	1.00 (0.94–1.05)	0.8486
		90–99 mmHg	15/1469	1.02	0.96 (0.90–1.02)	0.1682
		100–109 mmHg	6/759	0.79	1.01 (0.93–1.09)	0.8961
		≥110 mmHg	1/411	0.24	1.01 (0.91–1.13)	0.7976

Model adjusted for age, gender, BMI, heart rate, diabetes, hyperlipidemia, smoke, heart failure, myocardial infarction, stroke, chronic kidney disease, left main coronary artery disease, left ventricular ejection fraction, and creatinine clearance rate.

According to the RCS analysis, a similarly steep J-curve was observed (*p* for nonlinearity = 0.002) for DBP at admission concerning in-hospital mortality. Lower DBPs were linked to an increased risk of in-hospital mortality. The adjusted HR was 1.07 (95% CI: 1.01–1.14, *p* < 0.05) for patients with a DBP of 60–69 mmHg relative to the reference group of patients with a DBP in the 70–79 mmHg range (Table 5).

The analysis was adjusted for the covariates such as age, gender, BMI, heart rate, diabetes, hyperlipidemia, smoking and alcohol consumption history, heart failure, MI, chronic kidney disease, left main coronary artery disease, stroke, LVEF, and creatinine clearance rate.

### 3.3 The Association of SBP/DBP With 2-year HF

After adjusting for clinical baseline characteristics, a U-shaped curve was observed for the association between both SBP and DBP with the incidence of 2-year HF, with a nadir at 140/85 mmHg (all *p* for nonlinearity <0.001) (**Supplementary Fig. 1**).

Multivariate logistic regression models were used to investigate the association between SBP levels and 2-year heart failure. An elevated risk of 2-year HF was observed in the group with SBP ≤100 mmHg. In comparison to a reference group with an SBP of 120–130 mmHg, the adjusted odds ratios (ORs) were 2.01 for SBP <100 mmHg (95% CI: 1.64–2.46, *p* < 0.05) and 1.31 (95% CI: 1.06–1.63, *p* < 0.05) for SBP 100–109 mmHg, respectively. Compared to the reference group with a DBP of 70–79 mmHg, the adjusted ORs were 1.93 (95% CI: 1.56–2.38, *p* < 0.05) for DBP below 60 mmHg and 1.21 (95% CI: 1.02–1.44, *p* < 0.05) for DBP 60–69 mmHg, respectively. However, no statistically significant differences were observed for the higher SBP/DBP levels.

### 3.4 The Levels of SBP/DBP and All-cause Mortality and MACCEs

Across the entire cohort, an analysis of 2-year MACCEs revealed that 4.7% of individuals experienced all-cause mortality, 2.2% experienced recurrent MI, and 1.6% experienced stroke within 2 years. Both SBP and DBP showed a similar U-shaped relationship with the risk of MACCEs, with a nadir at 136/83 mmHg (**Supplementary Fig. 2**). A comparable pattern in the correlation with all-cause mortality showed the lowest point at 138/84 mmHg (**Supplementary Fig. 3**). For 2-year stroke events, the 95% confidence interval included a HR of 1.0 at any level of DBP (**Supplementary Fig. 4**). High SBP levels were linked to a greater risk of stroke despite a notably wide 95% confidence interval. Additionally, for 2-year recurrent MI in any group, the 95% confidence interval included a HR of 1.0 for all levels of both SBP and DBP (**Supplementary Fig. 5**).

### 3.5 The Levels of SBP/DBP and 2-year Severe Bleeding

A non-linear relationship in the form of a U-shape curve exists between SBP/DBP and the incidence of 2-year severe bleeding events was identified in the entire cohort (all *p* for nonlinearity < 0.001) (**Supplementary Fig. 6**). The risk of 2-year severe bleeding increased in cases of low BP for both SBP and DBP, with the lowest risk occurring at 138/84 mmHg. The adjusted HR for 2-year severe bleeding events was 1.06 (95% CI: 1.00–1.13, *p* < 0.05) in the groups with an admission DBP of 60–69 mmHg compared to the reference group with a DBP of 70–79 mmHg. Furthermore, the adjusted HRs for SBP <100 mmHg and 100–109 mmHg were 1.10 (95% CI: 1.02–1.19, *p* < 0.05) and 1.08 (95% CI: 1.00–1.16, *p* = 0.0537), respectively (**Supplementary Fig. 6**).

### 3.6 Subgroup Analyses

Age, gender, and diabetes were identified as prognostic factors for STEMI in the Cox regression analysis. Accordingly, patients were categorized into the younger group (≤65 years) and the older group (>65 years) for subgroup analysis. The association between SBP and in-hospital mortality within these two groups was non-linear (all *p* < 0.001). However, a nonlinear trend was not observed between DBP and in-hospital mortality in the older age group. The performance of curves for both groups is shown in **Supplementary Fig. 7**. In diabetes status and gender subgroups, similar nonlinear patterns, either J-shaped or U-shaped, were also observed except for the DBP group among females (**Supplementary Figs. 8,9**). Notably, the lower section had a steeper slope than the higher SBP section across all groups, indicating STEMI patients undergoing PCI suffered a greater risk from low SBP/DBP compared to high SBP/DBP.

## 4. Discussion

In this study involving 10,482 individuals from a large-scale Chinese cohort of consecutive STEMI patients undergoing PCI, a J-shaped association between SBP/DBP and in-hospital mortality was identified using RCS analysis. This J-curve relationship persisted as significant even after controlling for possible confounding variables. Our data indicate the following: (1) the optimal admission SBP/DBP for minimizing risk of in-hospital mortality is 157/94 mmHg; (2) Additionally, the RCS analysis reveals a J-shaped trend, where patients with lower admission SBP/DBP are associated with an elevated risk for 2-year incidence of HF, severe bleeding, 2-year MACCEs and all-cause mortality. However, no J-shaped association was found for recurrent MI. (3) As a categorical variable, the HRs for in-hospital mortality were significantly higher in groups with lower admission SBP/DBP levels compared to those with an admission SBP/DBP of 120–129/70–79 mmHg. This association remained even after adjusting for potential confounders. According to our knowledge, this is the first investigation of the relationship between SBP/DBP and various long-term adverse clinical outcomes in STEMI patients receiving PCI within a large Chinese population cohort.

The majority of previous studies were conducted as retrospective or post hoc analysis of randomized trials within hypertension cohorts. These studies often explore the association between BP and adverse endpoints but often fail to reach consistent results due to the variability in patient history and underlying fragility. A systematic review and meta-analysis, excluding ACS patients, confirmed the benefits of reducing SBP [5, 6]. However, it is crucial to recognize that lower BP is not universally better, particularly for certain patient populations like those with ACS. In the ONTARGET and TRANSCEND trials, patients treated for hypertension with an SBP <120 mmHg or a DBP <70 mmHg exhibited increased cardiovascular risk, compared to those patients admitted with an SBP ranging from 120–140 mmHg [24]. A J-shaped relationship between BP and all-cause mortality was also identified in patients with both hypertension and coronary artery disease (CAD), with the lowest risk occurring at 119/84 mmHg [16]. Notably, these studies did not include the population with known ACS, which may limit the generalizability of the findings to this specific population.

Limited studies have investigated the phenomenon of J-shaped or U-shaped curves in ACS individuals. Consistent with our observations, a J-shaped curve was shown for the relationship between admission SBP and 2-year cardiovascular mortality in elderly ACS patients [14]. The Acute Coronary Syndrome Israel Survey (ACSIS) revealed that patients admitted with an SBP below 110 mmHg experienced significantly higher all-cause mortality at both 7-day and 1-year compared to those with admission SBP (110–140 mmHg) [25]. Additionally, both lower and higher SBPs were related to increased risks for different outcomes in AMI patients, with the risk associated with lower SBP being greater than that of a higher SBP [26, 27, 28]. However, it is crucial to highlight that the aforementioned studies primarily concentrated on the relationship between BP and adverse clinical outcomes in ACS patients, without investigating the optimal BP levels related to the minimal risk of such adverse endpoints.

The optimal levels of BP in patients with ACS have not been adequately defined. Previous studies identified a J-shaped curve between SBP/DBP and cardiovascular risks, with a nadir of 136/85 mmHg in ACS patients [29]. Similarly, the J-shaped curve was observed for SBP in relation to AMI prognosis, with a nadir at 114 mmHg [26]. Our findings are concordant with previous studies indicating a J- or U-shaped curve between BP and adverse endpoints. However, our results diverge somewhat from earlier results. One possible explanation for this divergence could be differences in data quality, sample sizes and confounders adjusted in the models. Furthermore, few studies have investigated the direct impact of admission BP levels on both short-term and long-term outcomes in ACS patients receiving PCI. Unlike prior research focusing on hypertension cohorts or broader ACS populations, our study specifically targeted STEMI patients. Given the widespread availability of PCI, the majority of ACS patients can receive reperfusion therapy in a timely manner. Therefore, our study specifically examined STEMI patients undergoing PCI and also found that the J- or U- shaped curve phenomenon was not alleviated by reperfusion therapy.

Both lower and higher SBP are associated with increased risks for the prognosis of AMI, with a lower SBP creating a greater risk compared to that of a higher SBP [27, 28]. In contrast to studies conducted by US and European researchers, a small Japan–based study found an average admission SBP in the range of 141–159 mmHg, while ventricular rupture–related deaths were more frequently observed in both the group with SBP ≥160 mmHg and the group with lower SBP [27]. Furthermore, a higher SBP might increase the ventricular afterload, leading to diastolic dysfunction due to ventricular hypertrophy, myocardial damage induced by increasing oxygen consumption, and promotion of atherosclerotic plaque rupture [30, 31, 32].

Interestingly, paradoxical findings have emerged, suggesting that elevated SBP levels might have protective effects concerning short-term mortality and improved in-hospital prognosis among ACS patients [33, 34]. Patients with an extremely elevated SBP (>160 mmHg) on admission exhibited a reduced risk for the same endpoints [25]. Similarly, a study involving 3943 AMI patients from an Austrian tertiary care hospital found that an admission SBP of 120 mmHg or lower was linked to poorer outcomes compared to a normal SBP range of 121–140 mmHg. In contrast, the admission SBP (>160 mmHg) was related to the most favorable 1-year outcomes compared to a normal admission BP [35]. A study of 119,151 patients admitted for acute chest pain in the medical care unit (ICU) found that those with admission SBP in the highest quartile (>163 mmHg) had the lowest 1-year mortality compared to those in the second quartile (128–144 mmHg) [36]. SBP has historically been viewed as an indicator of peripheral resistance and cardiac output. Higher SBP may lead to improved prognosis in AMI patients, which may arise from the better-preserved cardiac functions and reduced myocardial damage in AMI patients. Another possible explanation is that the potentially beneficial effects of higher admission SBP might be attributed to the cardioprotective functions of anti-hypertension medications including ACEis and β receptor-blockers.

The J-shaped or U-shaped relationship, particularly the higher rate of adverse events at lower BP levels observed in this study can be attributed to several reasons: (i) Coronary reperfusion is influenced by two major factors: coronary arterial pressure and myocardial oxygen consumption [37]. A lower DBP often leads to decreased coronary reperfusion, and this phenomenon is more significant in coronary atherosclerotic patients with impaired coronary flow reserve [38]. In addition, it could be proposed that STEMI patients with reduced BP suffer poor systematic health status, overstimulation of the sympathetic nervous system and severe coronary microvascular dysfunction [39]. Consequently, the poorer outcomes in patients with reduced SBP/DBP might be attributed to the compromised reperfusion of the ischemic myocardium; (ii) Low BP may represent an inability to generate a hypertensive response, potentially reflecting an epiphenomenon resulting from comorbidity burden and frailty [40]. Regarding the J-shaped concept, it has raised concerns about reverse causality, suggesting that a low SBP/DBP might merely reflect the unhealthy condition of patients rather than directly causing worse cardiovascular outcomes. Increased risk at lower DBP demonstrated, at least in part, reverse causation due to arterial aging, stiffening, or other conditions that contribute to a lower DBP. However, the CLARIFY trial argued against this viewpoint, as the authors excluded certain conditions that affect life expectancy and other serious diseases. Even after adjusting for factors like heart failure, peripheral artery disease, and specific baseline characteristics, the link between low SBP/DBP and a higher risk of cardiovascular events remains [17]. (iii) Reduced SBP/DBP might also be an epiphenomenon of damaged cardiac function [41, 42]. Nonetheless, the study indicated that low DBP remained a significant predictor of adverse events, even after adjusting for left ventricular function [43]. Although different studies have identified varying BP thresholds based on different demographic characteristics, the findings consistently indicated that patients with a low SBP/DBP experienced an elevated risk of cardiovascular outcomes compared to reference groups.

Considering age and diabetes as key risk factors in hypertension patients, the management strategies for these populations should not be overlooked. For the elderly, particularly those with cardiovascular disease, arterial stiffness and multiple organ dysfunction are inevitable. However, there are no clear recommendations for very elderly hypertension patients. The very elderly (≥80 years) were recommended to control their BP to below 150/90 mmHg, while those aged 65–79 years were recommended to aim for a BP below 140/90 mmHg [44, 45]. Excessive reduction of BP might bring adverse outcomes, including reperfusion reduction in target organs and cognitive decline [46, 47]. For diabetic patients, strict BP control targeting 130/80 mmHg should be appropriate for patients with both diabetes and CAD [3, 48, 49]. Thus, when initiating BP management strategies for these populations, a comprehensive assessment of risk factors should be conducted. Dynamic BP monitoring and real-time drug adjustment are expected to avoid adverse clinical outcomes associated with excessive BP reduction. Further research should focus on the elderly and diabetic population to explore the optimal management strategy and avoid being extrapolated directly to these populations without caution. There is an urgent need for a specific BP-targeted threshold, particularly in high-risk populations.

## 5. Limitation

This study, sourced from the CAMI database, is a retrospective observational analysis, primarily involving patients from China. Therefore, the reported relationship in STEMI patients receiving PCI between admission SBP/DBP and the risk of endpoint events should not be extrapolated to other populations, such as those with different comorbidities or from different geographical regions. Despite the adjustments for numerous baseline confounders, our multivariable model failed to account for several unmeasured factors potentially affecting outcomes, including frailty, socioeconomic status, and mental health. The underlying mechanisms in the relationship between a low SBP/DBP and adverse clinical outcomes are multifactorial and not yet fully understood, necessitating caution when generalizing our observations. Furthermore, some patient data were not fully recorded, potentially influencing the validity of the findings. Considering that the admission BP levels may have been influenced by analgesic drugs and vasoactive drugs, future studies should account for these medications and their timing as confounders in the adjusted models.

Additionally, in our studies, the nadir points of SBP/DBP for different clinical outcomes were considered preliminary exploratory results. Although these findings could provide initial clues regarding the relationship between SBP/DBP and various clinical outcomes, further research is needed in different populations to confirm the generalizability and reliability of our findings. Moreover, we observed wide confidence intervals appeared near some SBP/DBP values, indicating greater uncertainties in risk estimates for adverse clinical outcomes at these BP values. As BP levels change, the width of the confidence interval may also vary, reflecting the instability or uncertainty of the risk estimates in these regions. In future studies, the accuracy of risk prediction could be improved by increasing the sample size and refining the group criteria.

## 6. Conclusion

In conclusion, our observational study of admitted STEMI patients undergoing PCI revealed a J-shaped relationship between admission SBP/DBP and in-hospital mortality risk with the lowest risk at 157/94 mmHg. The optimal values fluctuated around 140/85 mmHg in the relationship between admission SBP/DBP and long-term outcomes (2-year heart failure, MACCEs, and all-cause death). Further studies will focus on several key areas. First, multicenter, prospective cohort studies will be initiated to assess the effects of different types of hypertension, such as permissive hypertension and resistant hypertension on adverse endpoints like all-cause mortality, cardiovascular outcomes and cardiovascular-kidney-metabolic syndrome. Additionally, it is worth exploring the association between long-term BP variation and adverse clinical outcomes through advanced wearable devices. Finally, with the continued advancement of artificial intelligence, the analysis of large-scale population data on BP can be facilitated through artificial intelligence, machine learning and other advanced techniques for personalized and automatic identification, classification, and prediction.

## Availability of Data and Materials

The data supporting the findings of this study are available at https://www.chictr.org.cn/ with the registration number ChiCTR-ONC-12002636. However, the data is not publicly accessible at the moment. It can be obtained from the author, Yuejin Yang (yangyj_fw@126.com), upon reasonable request.

## Supplementary Material

Supplementary material associated with this article can be found, in the online version, at https://doi.org/10.31083/RCM33512.
