# Supplementary material for: Association Between Admission Blood Pressure and In-hospital Mortality and Long-term Mortality of Patients With ST-elevation Myocardial Infarction Undergoing Percutaneous Coronary Intervention: A China Acute Myocardial Infarction Registry Study
Source: Rev Cardiovasc Med. 2025 Aug 30;26(8):33512. doi: 10.31083/RCM33512 (PMC12415734; doi:10.31083/RCM33512)
Supplement: Supplementary file 1 [file 2153-8174-26-8-33512-s1.zip › Supplementary Table 1 and 2.docx]

**Table S1 Demographic and baseline characteristics of the patients by missing data status categories (missing vs non-missing).**

|  | Missing data status | |  |
| --- | --- | --- | --- |
|  | Missing | Non-Missing | P-value |
| N (total=10482) | 7209 | 3273 | - |
| Age (years) | 60.15±11.85 | 59.16±11.61 | <0.001 |
| Male sex | 5782(80.2%) | 2668(81.5%) | 0.1147 |
| BMI (kg/m^2^) | 24.36±3.07 | 24.38±2.93 | 0.7337 |
| Baseline SBP (mmHg) | 127.92±24.75 | 125.80±24.44 | <0.001 |
| Baseline DBP (mmHg) | 79.10±15.83 | 78.10±15.54 | 0.0026 |
| Heart rate (b.p.m.) | 76.53±16.71 | 75.17±16.84 | <0.001 |
| LVEF (%) | 53.86±10.26 | 53.70±9.87 | 0.4504 |
| Killip III/IV(%) | 381(5.3%) | 166(5.1%) | 0.6136 |
| Mean glucose（mmol/L） | 8.88±45.86 | 10.38±78.70 | 0.3139 |
| LDL-C（mmol/L） | 2.90±2.74 | 2.84±1.34 | 0.1583 |
| HDL-C（mmol/L） | 1.16±2.67 | 1.09±2.09 | 0.1810 |
| Total Cholesterol（mmol/L） | 4.62±1.38 | 4.58±1.76 | 0.3641 |
| Triglycerides（mmol/L） | 1.80±2.07 | 1.89±5.18 | 0.3571 |
| Hs-CRP（mg/L） | 15.77±28.31 | 16.89±28.47 | 0.2539 |
| NT-proBNP（fmmol/L） | 1409.2±2979.8 | 1353.7±2643.4 | 0.4973 |
| WBC (x10^9/L) | 10.65±3.56 | 10.46±3.49 | 0.0086 |
| PLT (x10^9/L) | 212.73±64.95 | 213.30±63.35 | 0.6778 |
| Serum creatinine (mmol/L) | 78.75±37.53 | 77.97±29.51 | 0.2535 |
| Previous MI | 355(5.3%) | 165(5.0%) | 0.5300 |
| TnI (ng/mL) | 37.68±53.53 | 39.84±63.47 | 0.2847 |
| History of heart failure | 65(1.0%) | 13(0.4%) | 0.0014 |
| Admission heart failure | 839(11.7%) | 313(9.6%) | 0.0013 |
| Admission cardiogenic Shock | 185(2.6%) | 112(3.4%) | 0.0173 |
| Admission cardiac arrest | 75(1.0%) | 32(1.0%) | 0.7583 |
| Peripheral vascular disease | 31(0.5%) | 12(0.4%) | 0.5195 |
| Hyperlipidemia | 444(7.3%) | 363(11.1%) | <0.001 |
| Diabetes | 1310(19.1%) | 570(17.4%) | 0.0436 |
| Prior stroke | 593(8.7%) | 200(6.1%) | <0.001 |
| COPD | 98(1.4%) | 41(1.3%) | 0.4785 |
| Haemoglobin (g/dL) | 139.78±19.66 | 139.98±18.41 | 0.6098 |
| ACEi/ARB | 371(5.8%) | 210(7.0%) | 0.0239 |
| Beta-blocker | 264(4.1%) | 179(6.0%) | <0.001 |
| Aspirin | 544(8.3%) | 282(9.3%) | 0.1191 |
| Clopidogrel | 226(3.4%) | 114(3.7%) | 0.4574 |
| Diuretics | 62(1.0%) | 23(0.8%) | 0.3690 |
| CCB | 487(7.6%) | 200(6.6%) | 0.0985 |

Continuous variables are medians with 25th and 75th percentiles. Abbreviations: BMI, Body Mass Index; SBP, Systolic blood pressure; DBP, diastolic blood pressure; LVEF, left ventricular ejection fraction; LDL-C, low-density lipoprotein cholesterol; HDL-C, low-density lipoprotein cholesterol; Hs-CRP, High-sensitivity C-reactive protein; NT-proBNP, N-terminal pro-brain natriuretic peptide; WBC, white blood cell; PLT, platelet; MI, myocardial infarction; TnI, Troponin I/T; COPD, Chronic Obstructive Pulmonary Disease; ACEi/ARB, angiotensin-converting enzyme inhibitor/angiotensin receptor blocker.

**Table S2 Demographic and baseline characteristics of the patients by missing data status categories (missing vs non-missing).**

|  | Non-Missing(n) | Missing(n) | Missing(%) |
| --- | --- | --- | --- |
| N (total=10482) | 10482 | - |  |
| Age (years) | 10415 | 67 | 0.64 |
| Male sex | 10482 | 0 | 0.00 |
| BMI (kg/m^2^) | 10139 | 343 | 3.27 |
| Baseline SBP (mmHg) | 10482 | 0 | 0.00 |
| Baseline DBP (mmHg) | 10482 | 0 | 0.00 |
| Heart rate (b.p.m.) | 10470 | 12 | 0.11 |
| LVEF (%) | 9106 | 1376 | 13.13 |
| Killip III/IV(%) | 10444 | 38 | 0.36 |
| Mean glucose（mmol/L） | 10105 | 377 | 3.60 |
| LDL-C（mmol/L） | 9676 | 806 | 7.69 |
| HDL-C（mmol/L） | 9687 | 795 | 7.58 |
| Total Cholesterol（mmol/L） | 9791 | 691 | 6.59 |
| Triglycerides（mmol/L） | 9895 | 587 | 5.60 |
| Hs-CRP（mg/L） | 3699 | 6783 | 64.71 |
| NT-proBNP（fmmol/L） | 5139 | 5343 | 50.97 |
| WBC (x10^9/L) | 10345 | 137 | 1.31 |
| PLT (x10^9/L) | 10338 | 144 | 1.37 |
| Serum creatinine (mmol/L) | 10287 | 195 | 1.86 |
| Previous MI | 9922 | 560 | 5.34 |
| TnI (ng/mL) | 3806 | 6676 | 63.69 |
| History of heart failure | 10030 | 452 | 4.31 |
| Admission heart failure | 10409 | 73 | 0.70 |
| Admission cardiogenic Shock | 10432 | 50 | 0.48 |
| Admission cardiac arrest | 10453 | 29 | 0.28 |
| Peripheral vascular disease | 10066 | 416 | 3.97 |
| Hyperlipidemia | 9397 | 1085 | 10.35 |
| Diabetes | 10141 | 341 | 3.25 |
| Prior stroke | 10109 | 373 | 3.56 |
| COPD | 10051 | 467 | 4.46 |
| Haemoglobin (g/dL) | 10335 | 147 | 1.40 |
| ACEi/ARB | 9397 | 1085 | 10.35 |
| Beta-blocker | 9436 | 1046 | 9.98 |
| Aspirin | 9573 | 909 | 8.67 |
| Clopidogrel | 9612 | 870 | 8.30 |
| Diuretics | 9526 | 956 | 9.12 |
| CCB | 9433 | 1049 | 10.01 |

Continuous variables are medians with 25th and 75th percentiles. Abbreviations: BMI, Body Mass Index; SBP, Systolic blood pressure; DBP, diastolic blood pressure; LVEF, left ventricular ejection fraction; LDL-C, low-density lipoprotein cholesterol; HDL-C, low-density lipoprotein cholesterol; Hs-CRP, High-sensitivity C-reactive protein; NT-proBNP, N-terminal pro-brain natriuretic peptide; WBC, white blood cell; PLT, platelet; MI, myocardial infarction; TnI, Troponin I/T; COPD: Chronic Obstructive Pulmonary Disease; ACEi/ARB, angiotensin-converting enzyme inhibitor/angiotensin receptor blocker.
