# Supplementary material for: Association Between Admission Blood Pressure and In-hospital Mortality and Long-term Mortality of Patients With ST-elevation Myocardial Infarction Undergoing Percutaneous Coronary Intervention: A China Acute Myocardial Infarction Registry Study
Source: Rev Cardiovasc Med. 2025 Aug 30;26(8):33512. doi: 10.31083/RCM33512 (PMC12415734; doi:10.31083/RCM33512)
Supplement: Supplementary file 1 [file 2153-8174-26-8-33512-s1.zip › supplementary figures1-9.docx]

**
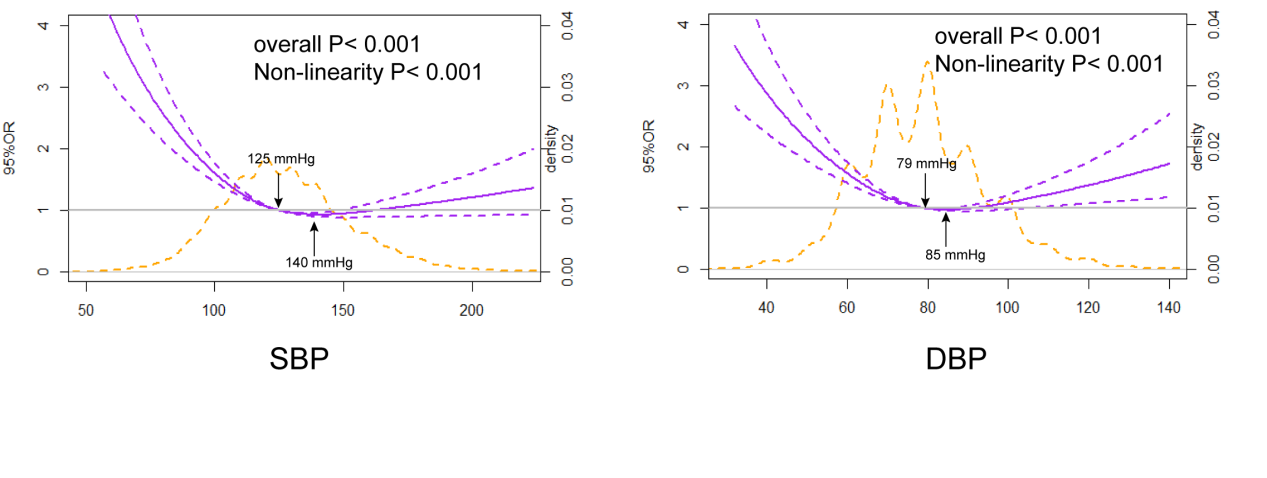

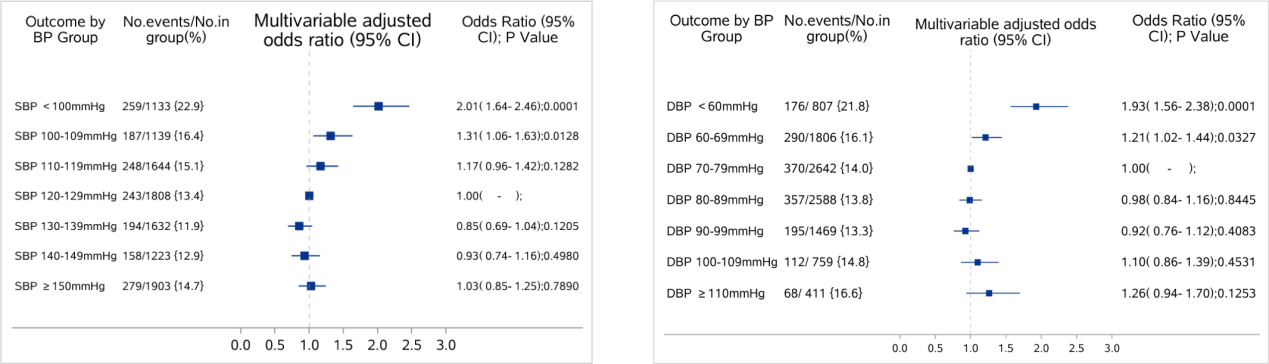
** **Supplementary Fig. 1. 2-year heart failure.**

**Supplementary Fig. 2. 2-year MACCEs.**

**
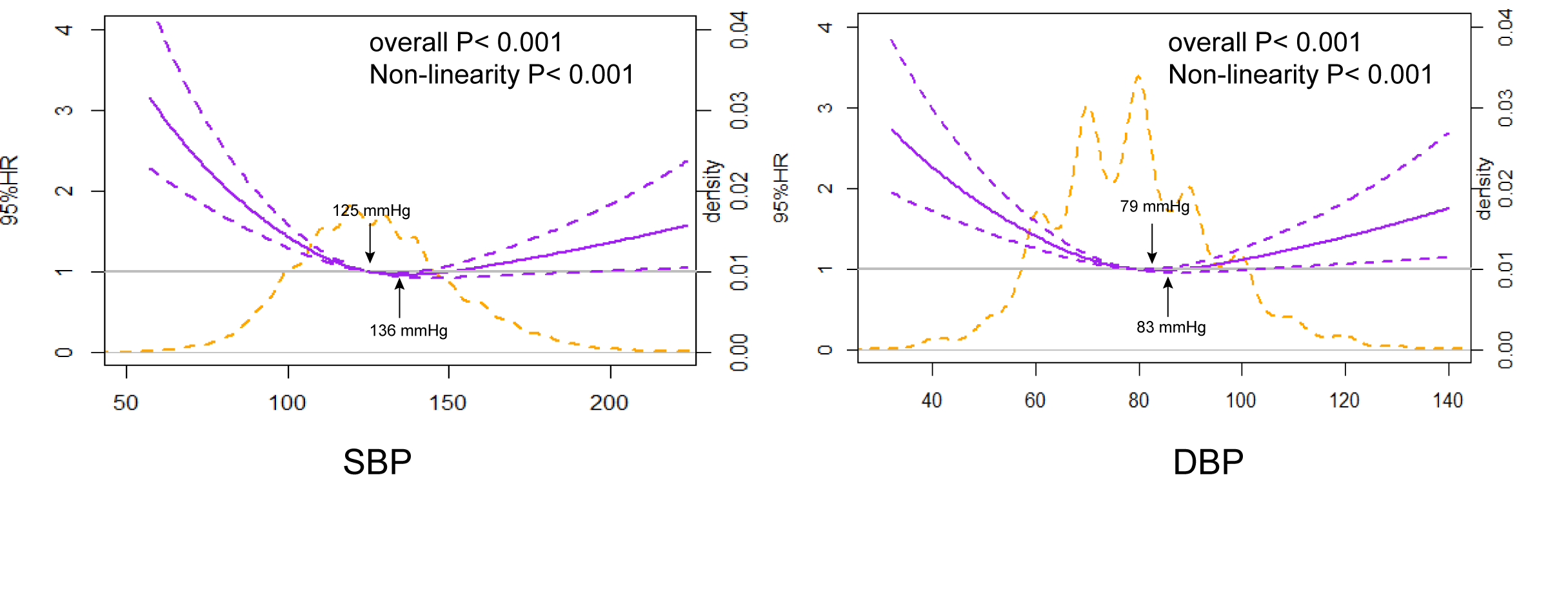
**

**
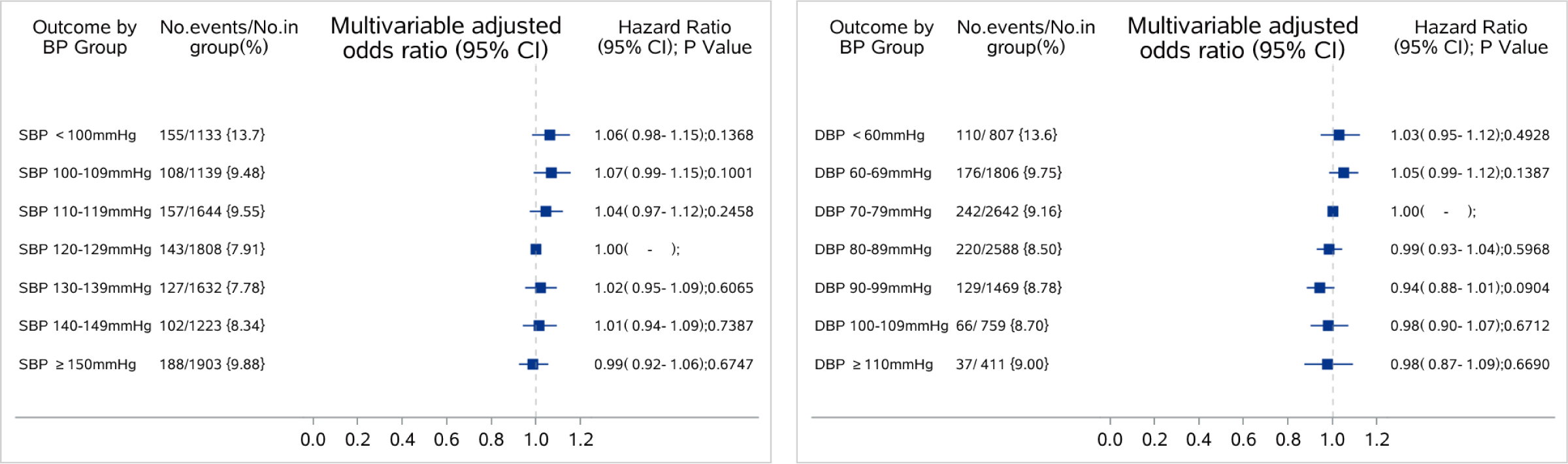
**

**Supplementary Fig. 3. 2-year all-cause mortality.**

**
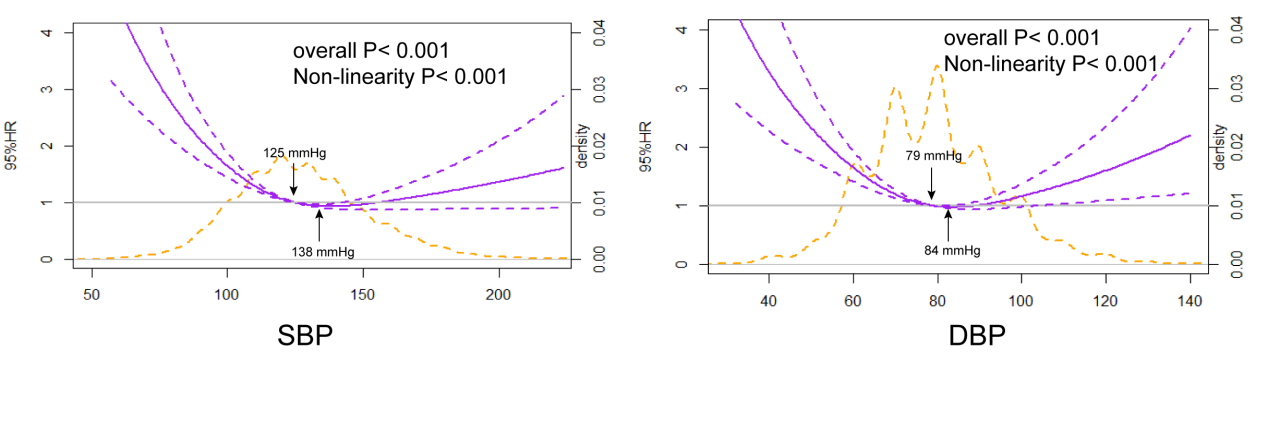
**

**
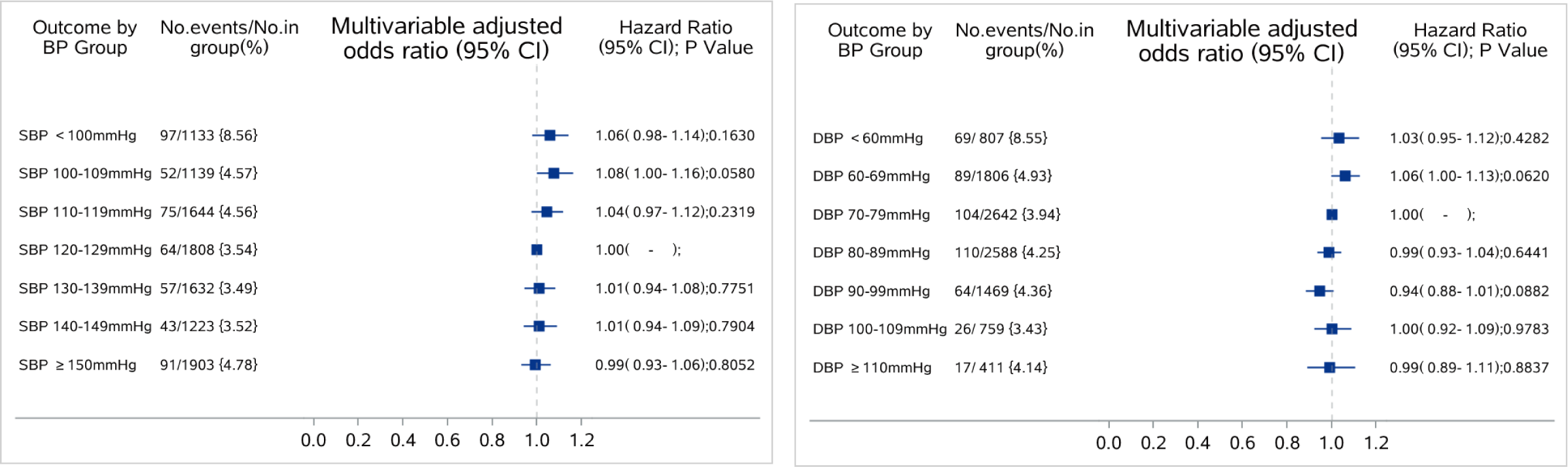
**

**Supplementary Fig. 4. 2-year stroke.**

**
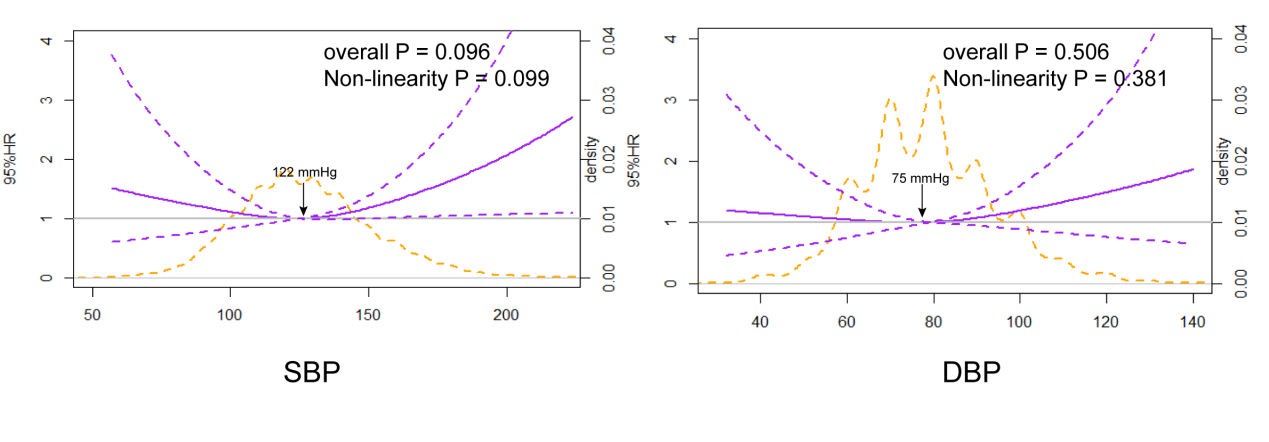
**

**
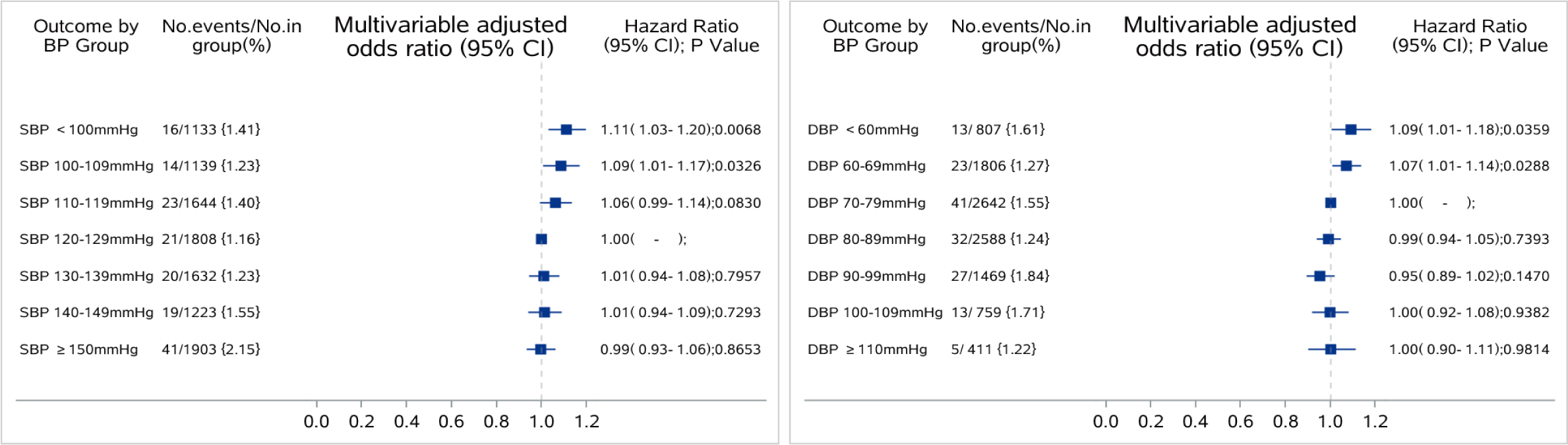
**

**Supplementary Fig. 5. 2-year recurrent myocardial infarction.**

**
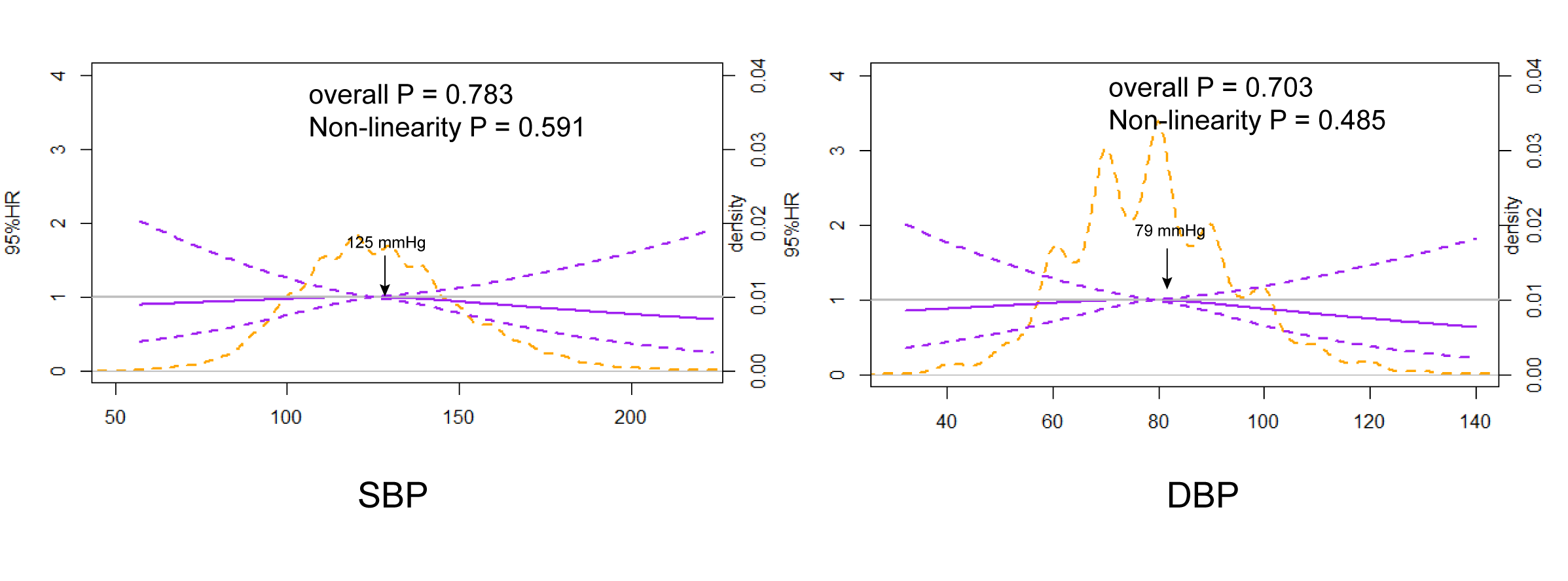
**

**
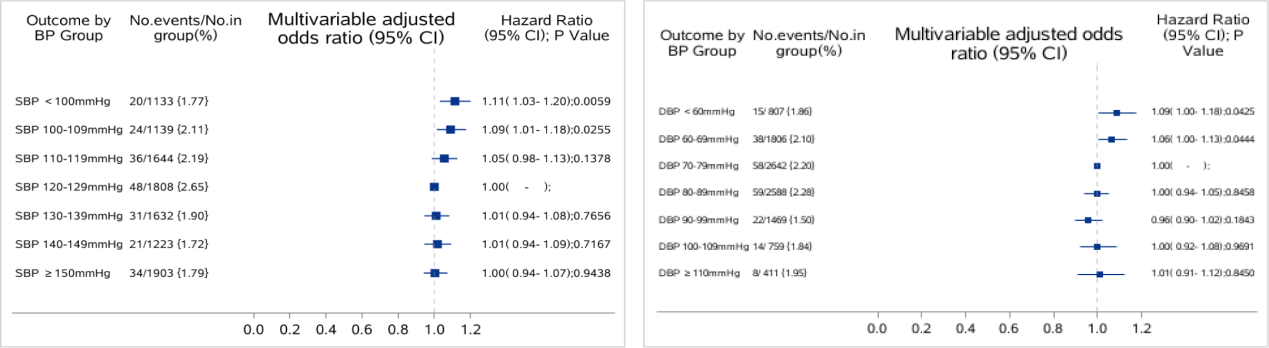
**

**Supplementary Fig. 6. 2-year severe bleeding.**

**
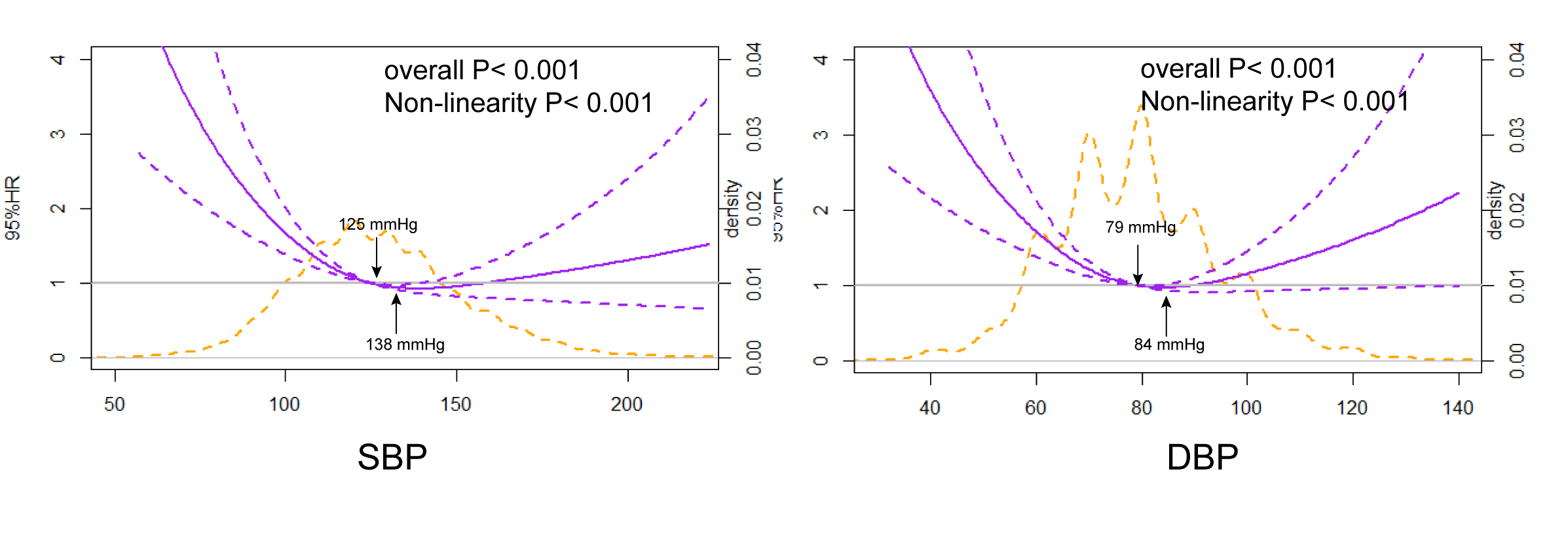
**

**
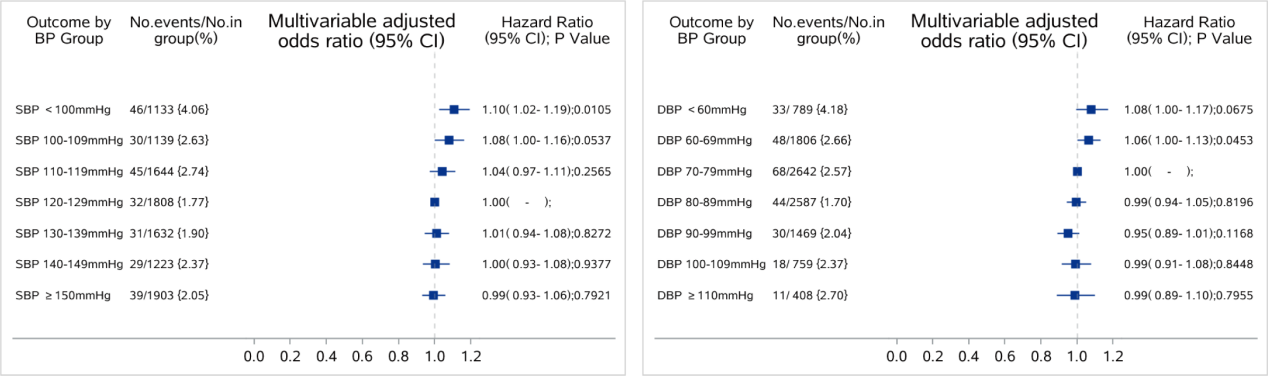
**

**Supplementary Fig. 7. age < 65 and age ≥ 65.**

**
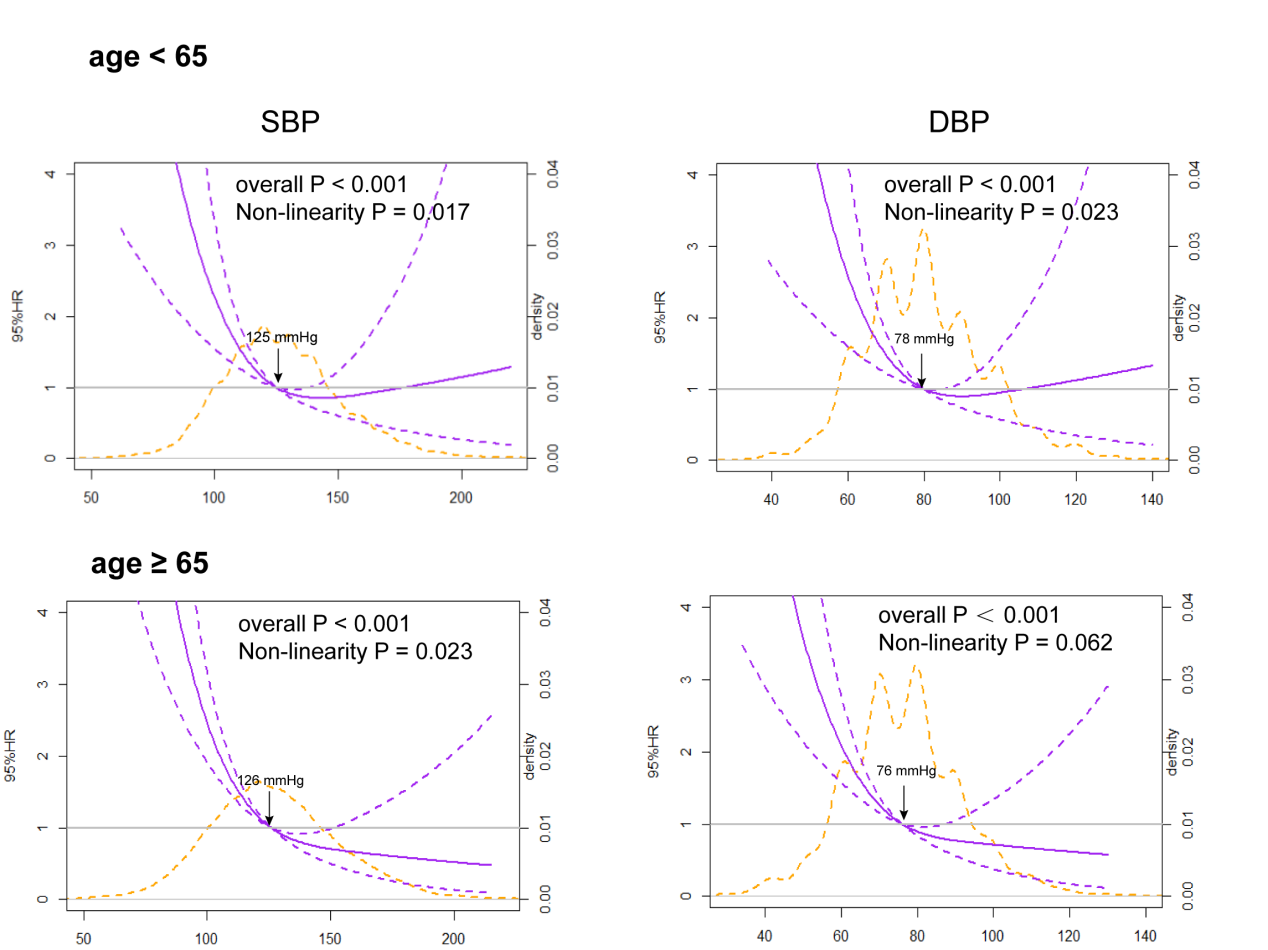
**

**Supplementary Fig. 8. Male and female.**

**
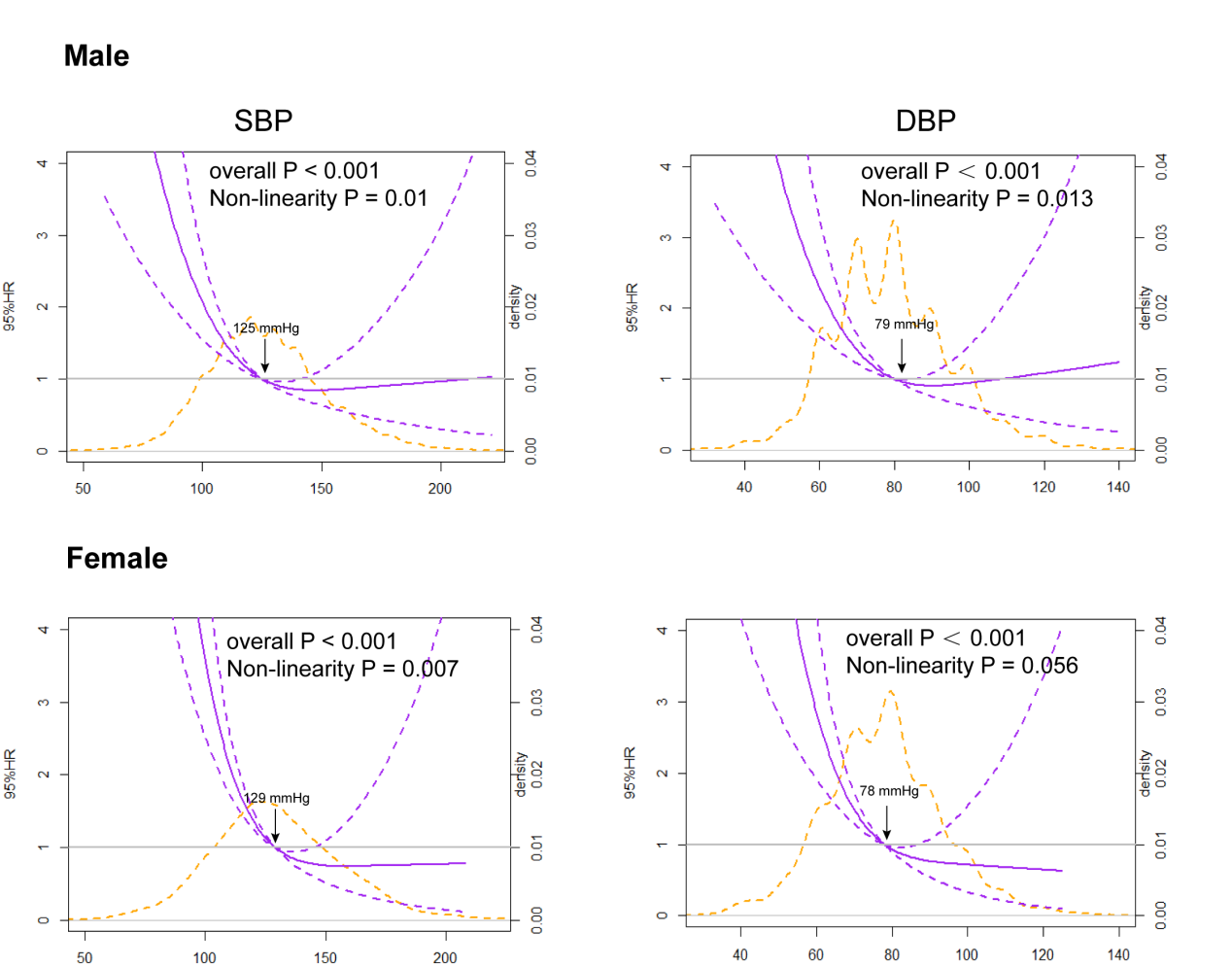
**

**Supplementary Fig. 9. Diabetes and non-diabetes.**

**
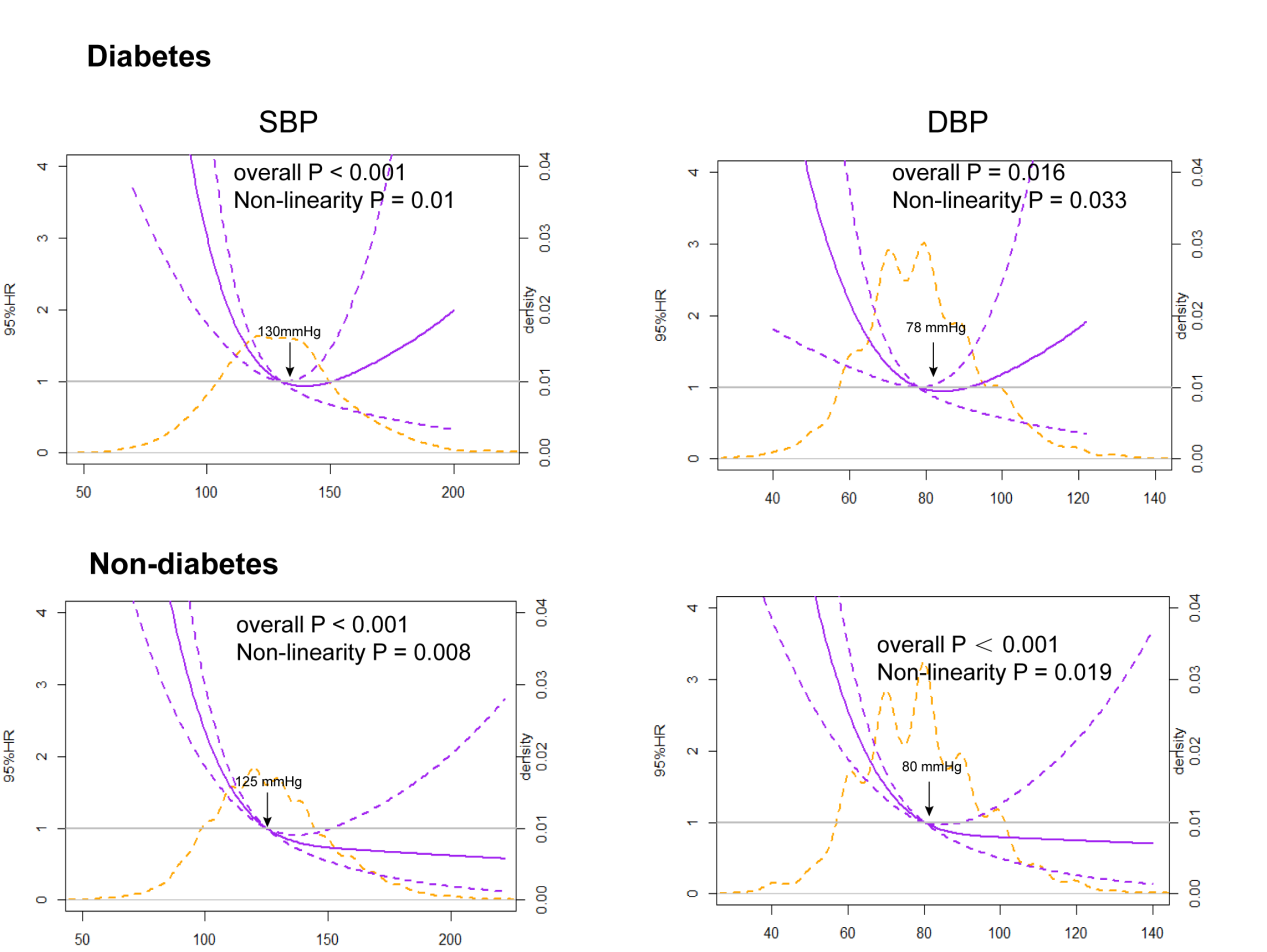
**
