# Supplementary figures and images for: Association Between Admission Blood Pressure and In-hospital Mortality and Long-term Mortality of Patients With ST-elevation Myocardial Infarction Undergoing Percutaneous Coronary Intervention: A China Acute Myocardial Infarction Registry Study
Source: Rev Cardiovasc Med. 2025 Aug 30;26(8):33512. doi: 10.31083/RCM33512 (PMC12415734; doi:10.31083/RCM33512)

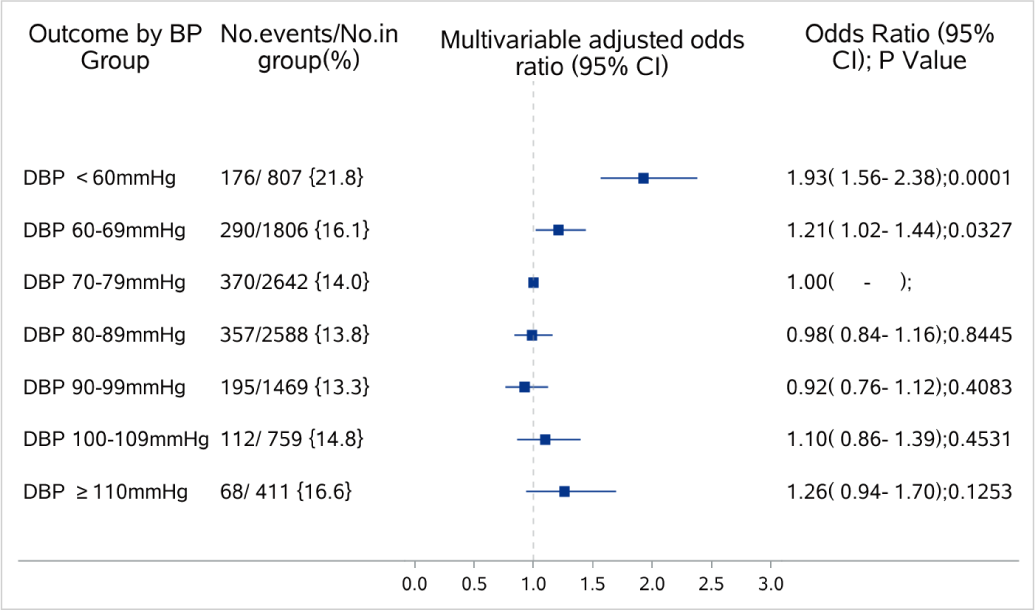

Supplement: Supplementary file 1 [file 2153-8174-26-8-33512-s1.zip › Supplementary Fig. 1/2-year HF-forest plot-DBP.tiff]

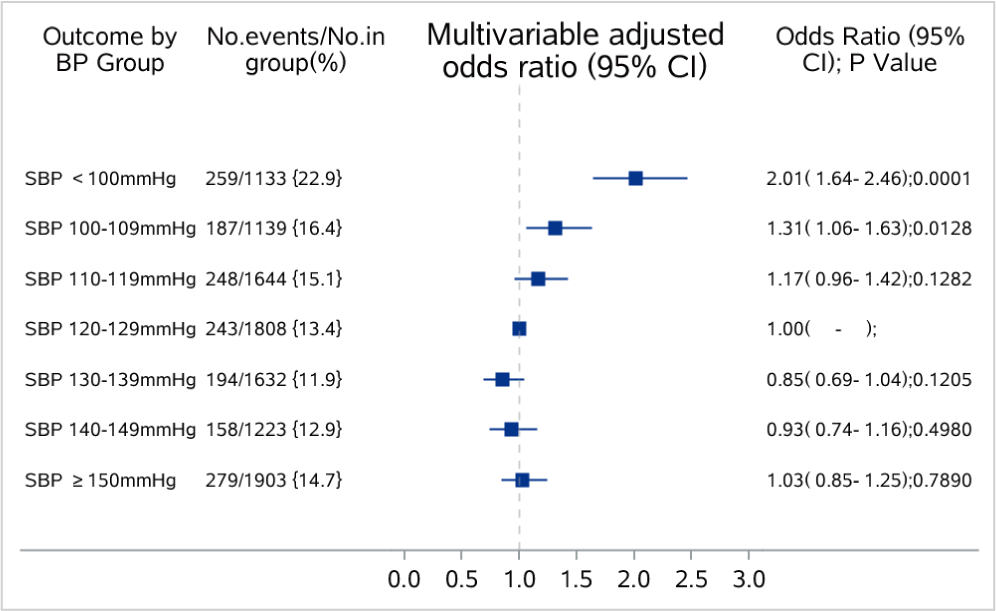

Supplement: Supplementary file 1 [file 2153-8174-26-8-33512-s1.zip › Supplementary Fig. 1/2-year HF-forest plot-SBP.tiff]

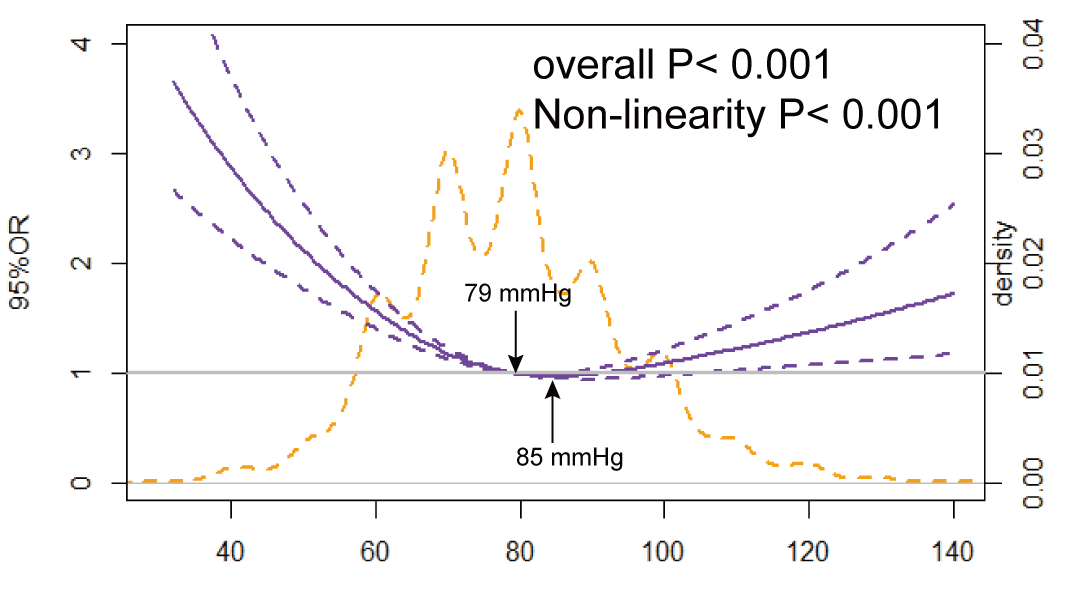

Supplement: Supplementary file 1 [file 2153-8174-26-8-33512-s1.zip › Supplementary Fig. 1/2-year HF-RCS-DBP.tiff]

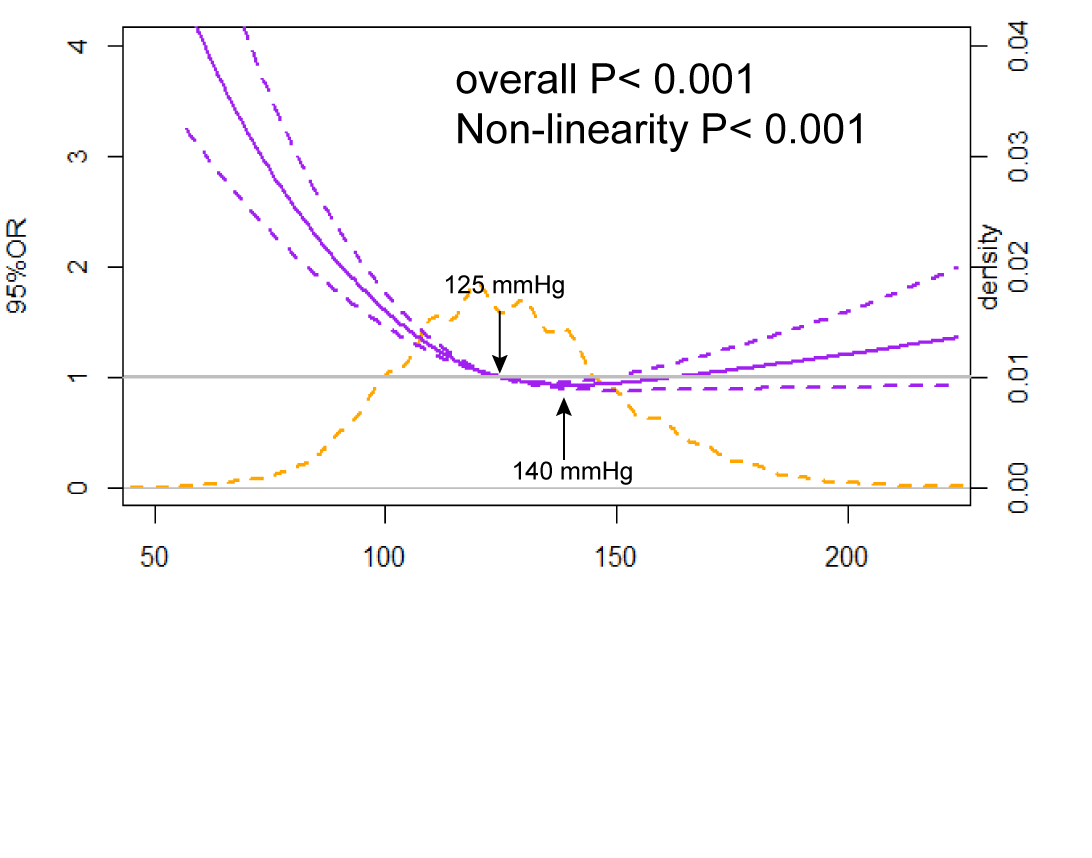

Supplement: Supplementary file 1 [file 2153-8174-26-8-33512-s1.zip › Supplementary Fig. 1/2-year HF-RCS-SBP.tiff]

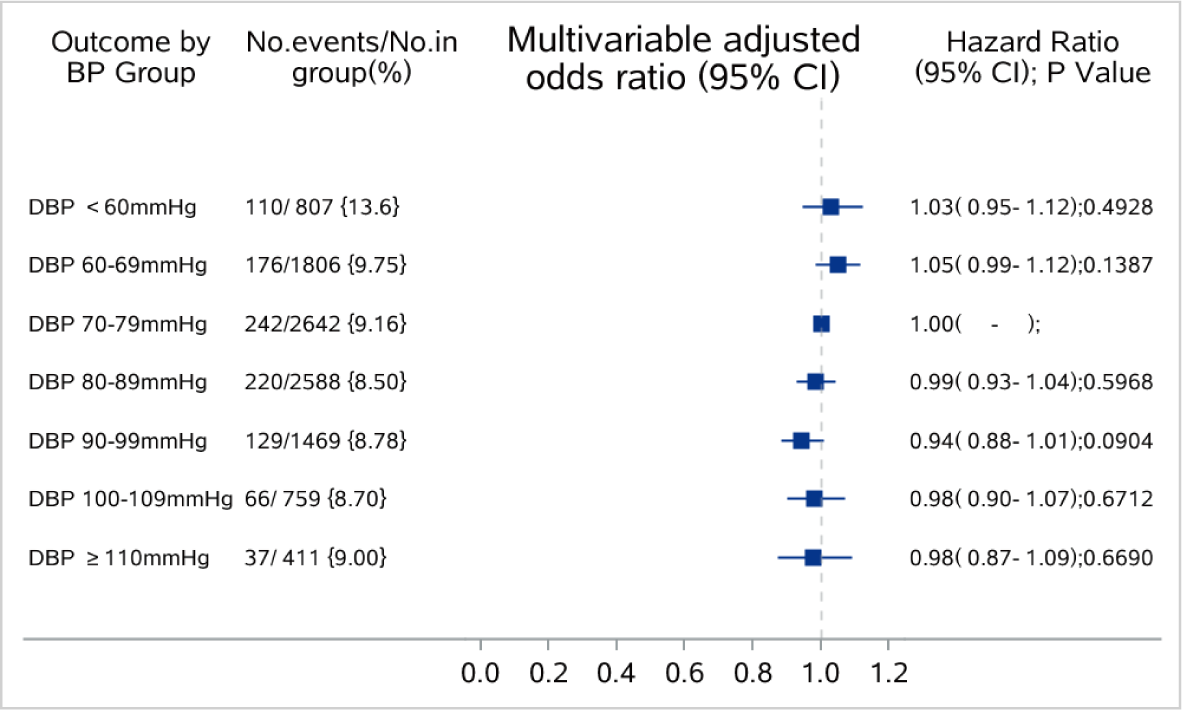

Supplement: Supplementary file 1 [file 2153-8174-26-8-33512-s1.zip › Supplementary Fig. 2/MACCE-forest plot-DBP.tiff]

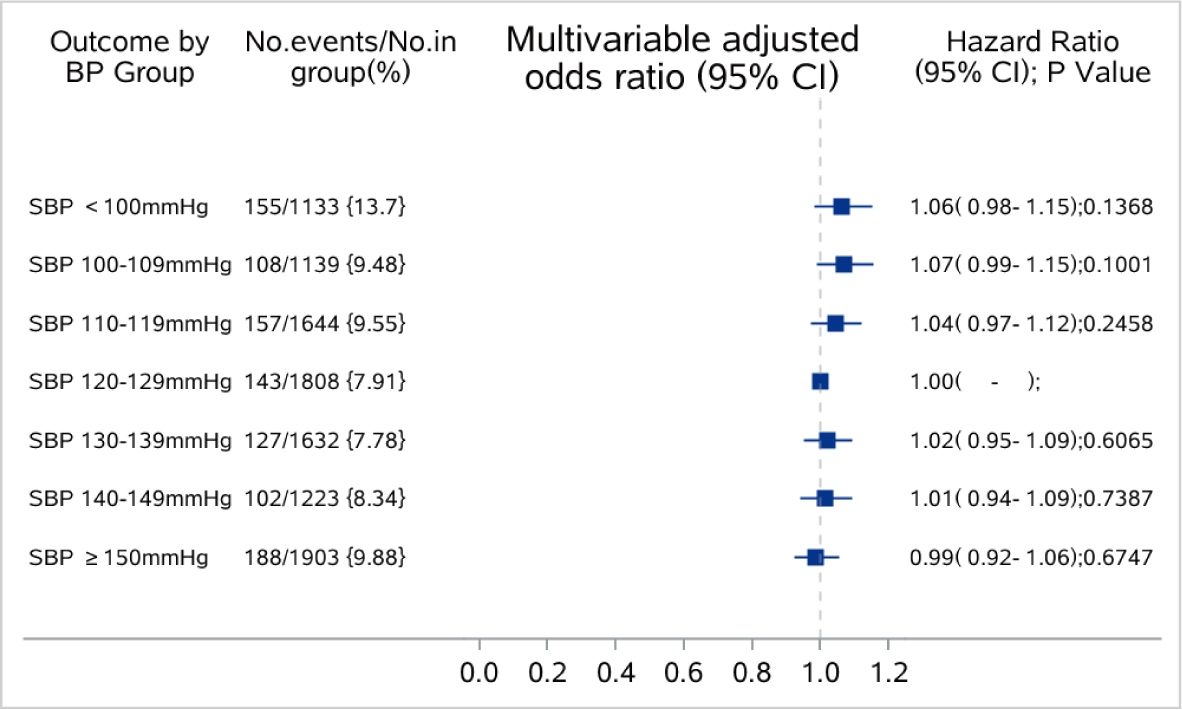

Supplement: Supplementary file 1 [file 2153-8174-26-8-33512-s1.zip › Supplementary Fig. 2/MACCE-forest plot-SBP.tiff]

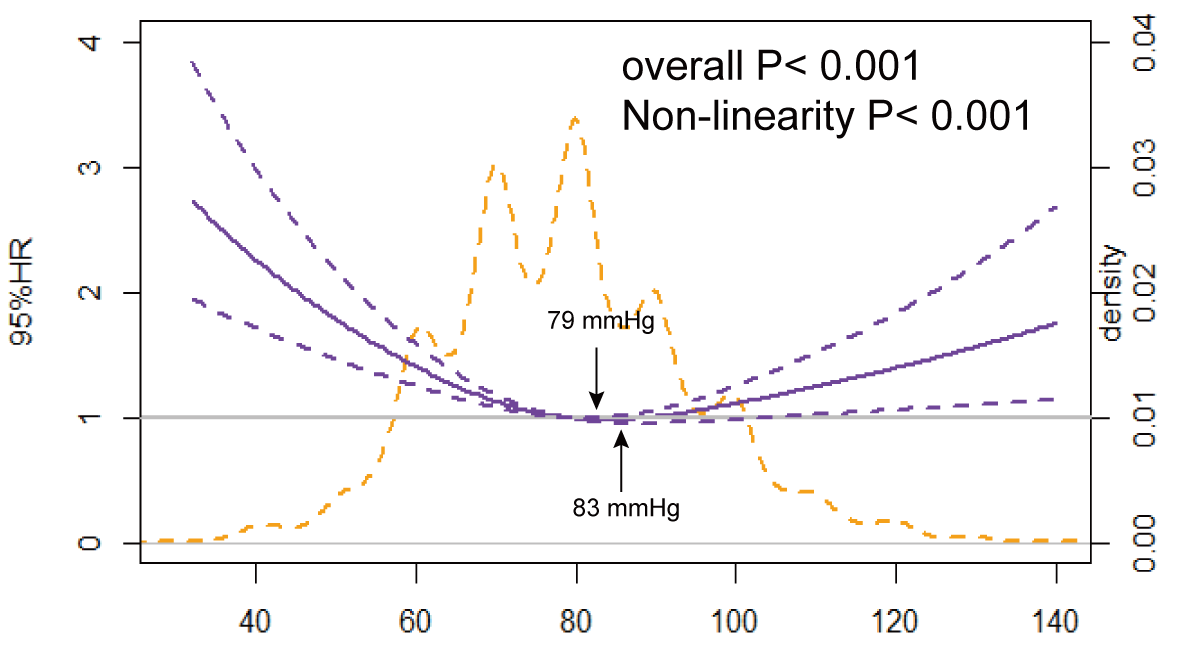

Supplement: Supplementary file 1 [file 2153-8174-26-8-33512-s1.zip › Supplementary Fig. 2/MACCE-RCS-DBP.tiff]

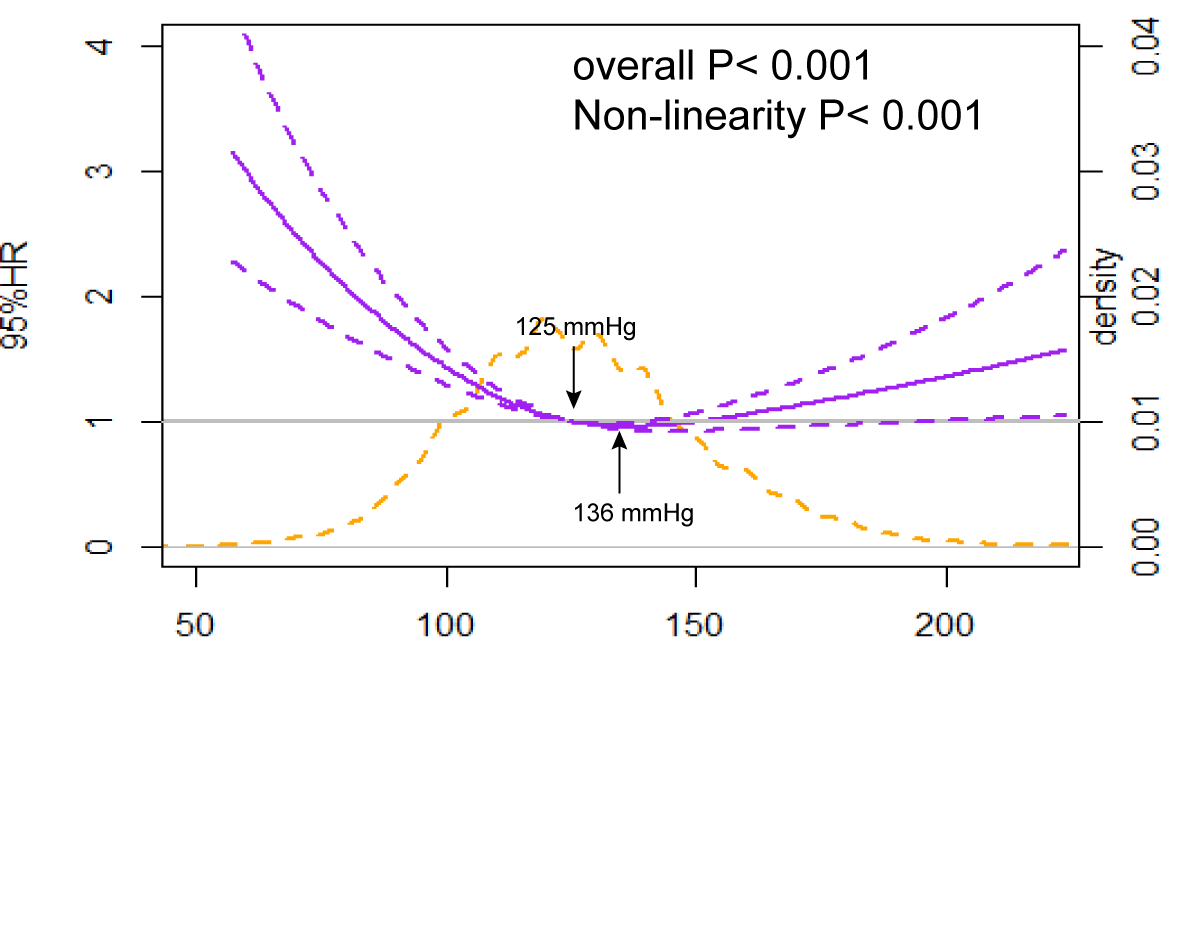

Supplement: Supplementary file 1 [file 2153-8174-26-8-33512-s1.zip › Supplementary Fig. 2/MACCE-RCS-SBP.tiff]

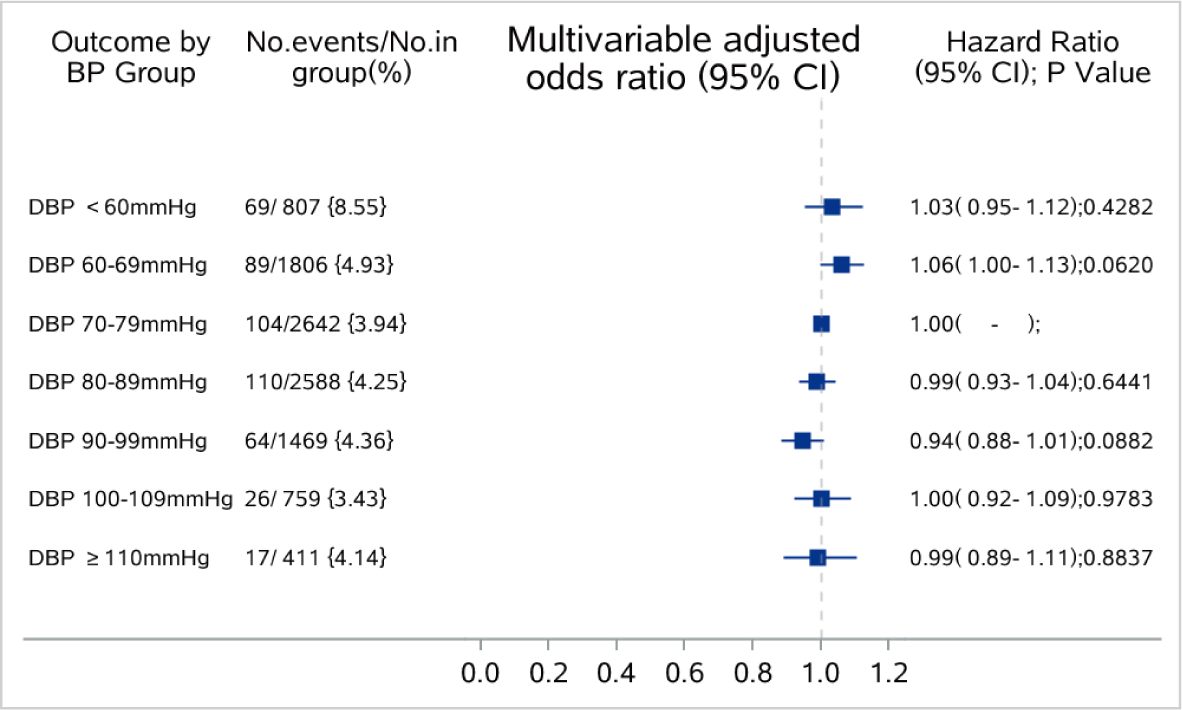

Supplement: Supplementary file 1 [file 2153-8174-26-8-33512-s1.zip › Supplementary Fig. 3/all-cause mortality-forest plot-DBP.tiff]

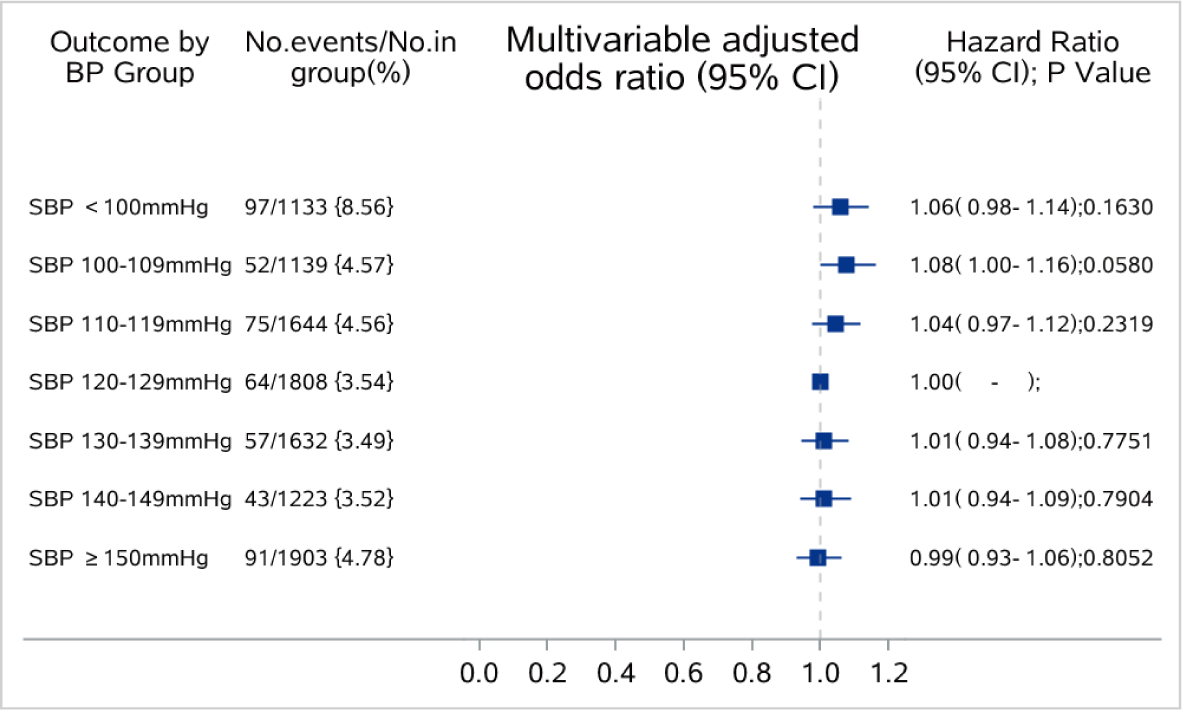

Supplement: Supplementary file 1 [file 2153-8174-26-8-33512-s1.zip › Supplementary Fig. 3/all-cause mortality-forest plot-SBP.tiff]

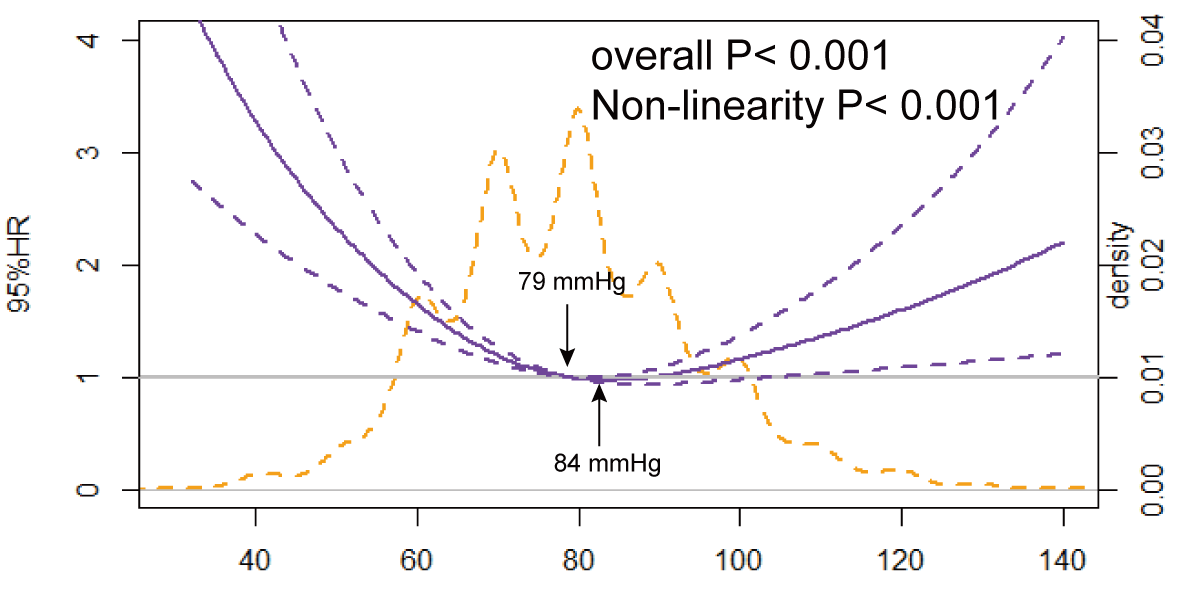

Supplement: Supplementary file 1 [file 2153-8174-26-8-33512-s1.zip › Supplementary Fig. 3/all-cause mortality-RCS-DBP.tiff]

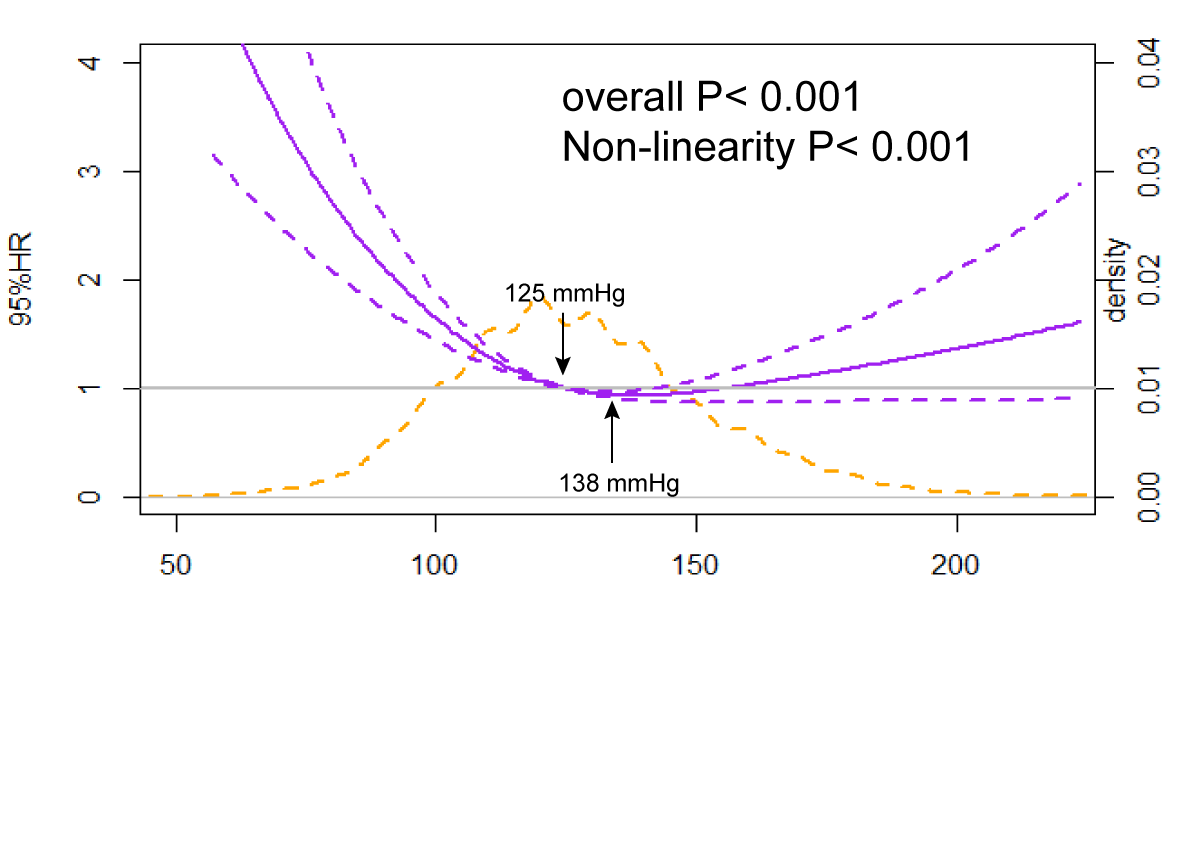

Supplement: Supplementary file 1 [file 2153-8174-26-8-33512-s1.zip › Supplementary Fig. 3/all-cause mortality-RCS-SBP.tiff]

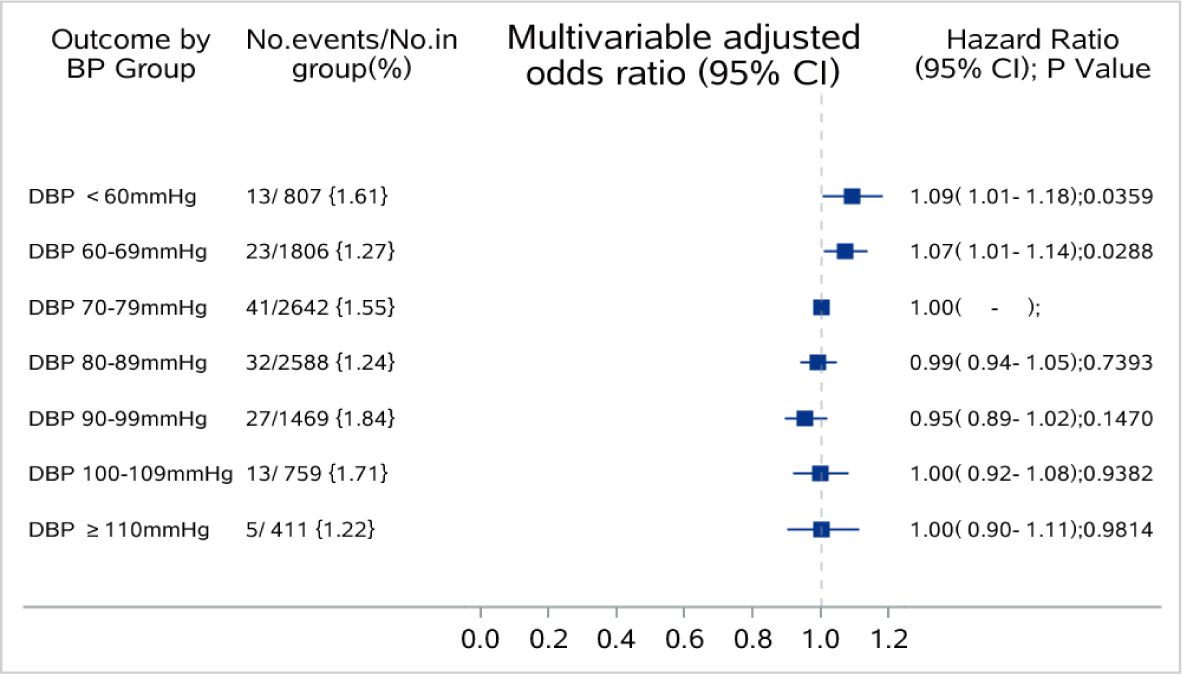

Supplement: Supplementary file 1 [file 2153-8174-26-8-33512-s1.zip › Supplementary Fig. 4/stroke-forest plot-DBP.tiff]

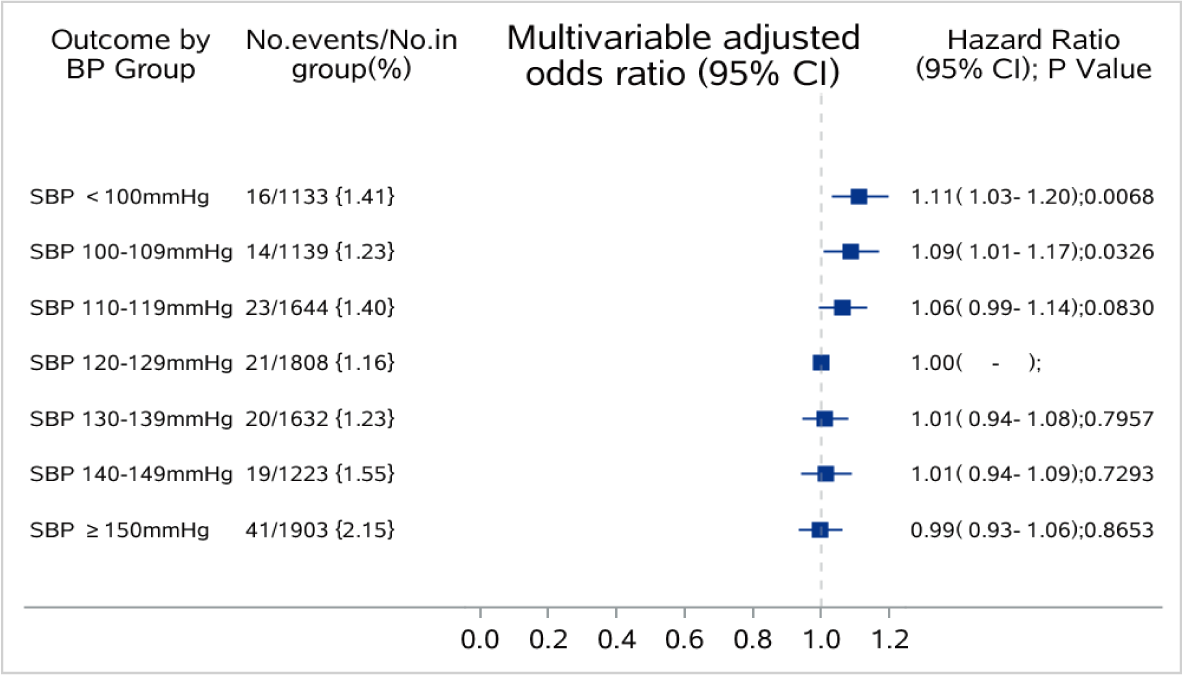

Supplement: Supplementary file 1 [file 2153-8174-26-8-33512-s1.zip › Supplementary Fig. 4/stroke-forest plot-SBP.tiff]

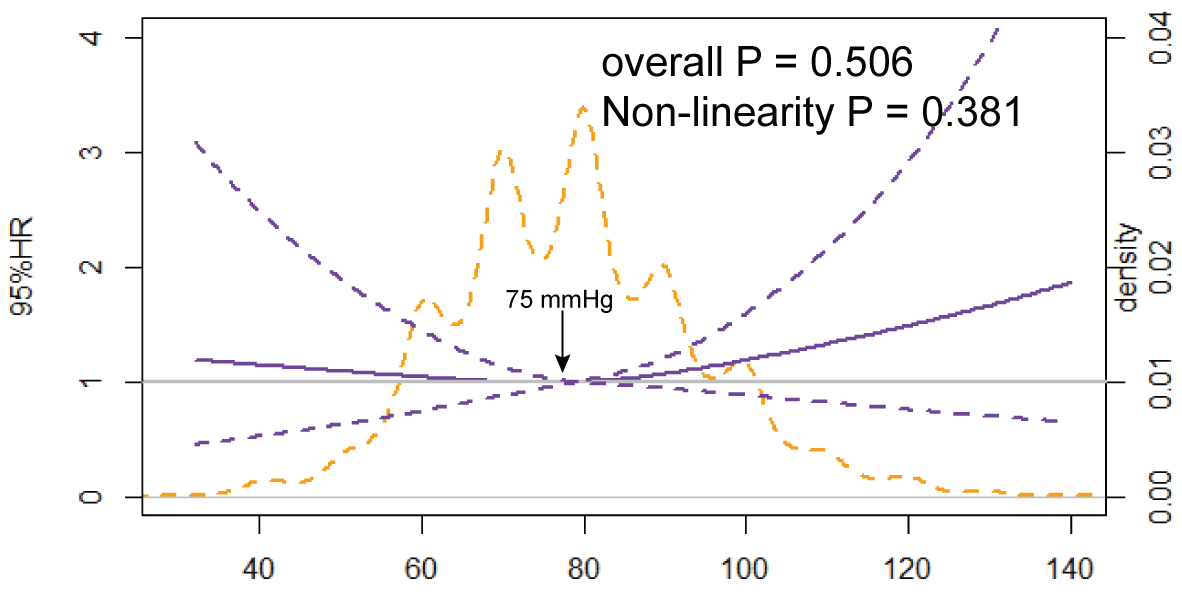

Supplement: Supplementary file 1 [file 2153-8174-26-8-33512-s1.zip › Supplementary Fig. 4/stroke-RCS-DBP.tiff]

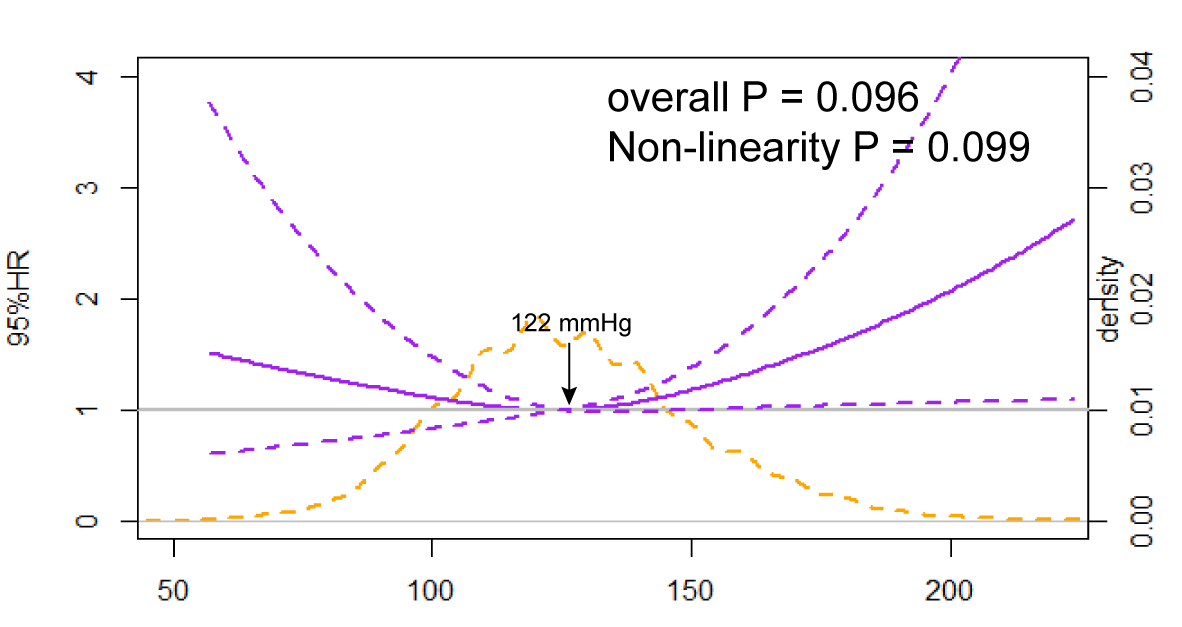

Supplement: Supplementary file 1 [file 2153-8174-26-8-33512-s1.zip › Supplementary Fig. 4/stroke-RCS-SBP.tiff]

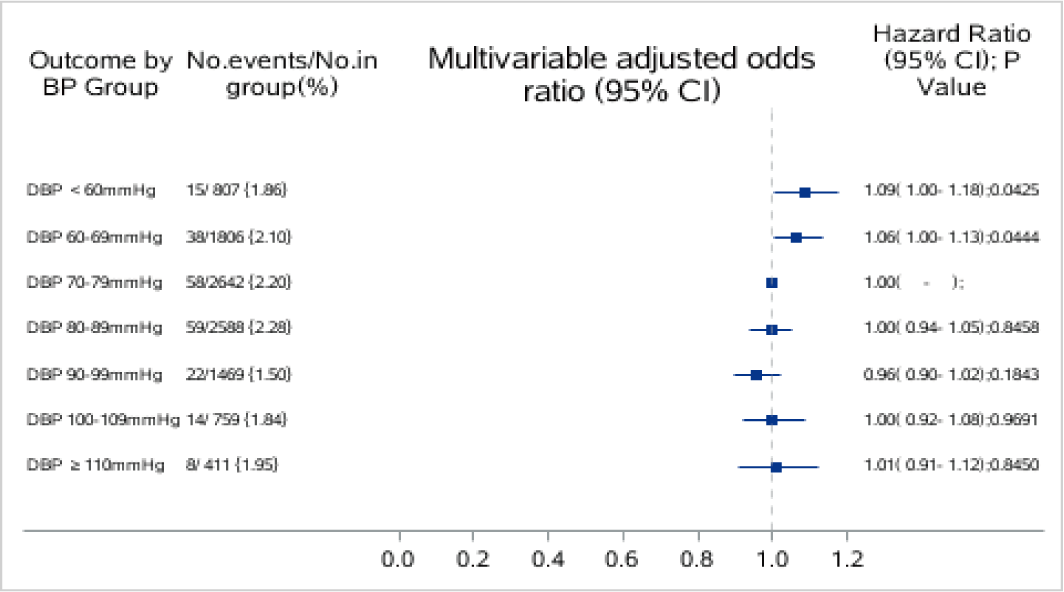

Supplement: Supplementary file 1 [file 2153-8174-26-8-33512-s1.zip › Supplementary Fig. 5/recurrent MI-forest plot-DBP.tiff]

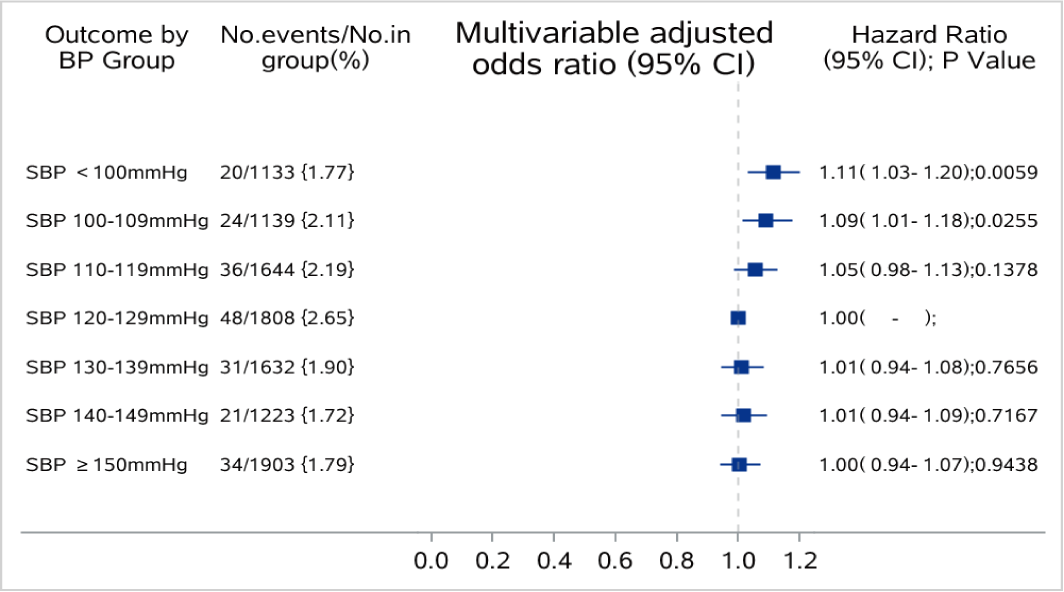

Supplement: Supplementary file 1 [file 2153-8174-26-8-33512-s1.zip › Supplementary Fig. 5/recurrent MI-forest plot-SBP.tiff]

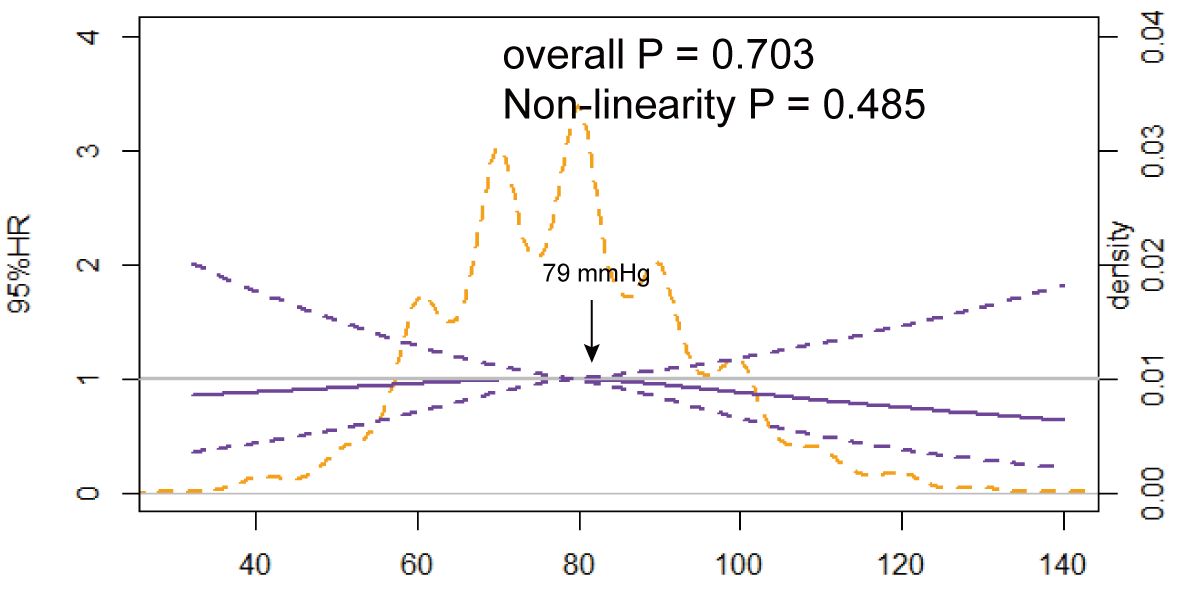

Supplement: Supplementary file 1 [file 2153-8174-26-8-33512-s1.zip › Supplementary Fig. 5/recurrent MI-RCS-DBP.tiff]

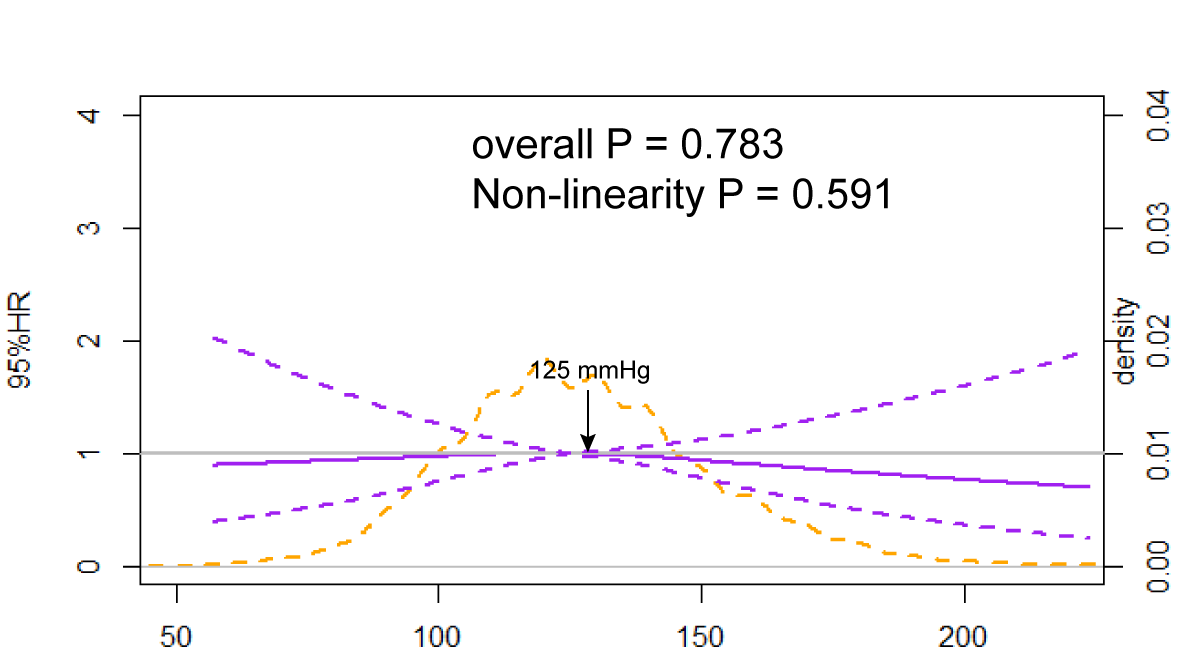

Supplement: Supplementary file 1 [file 2153-8174-26-8-33512-s1.zip › Supplementary Fig. 5/recurrent MI-RCS-SBP.tiff]

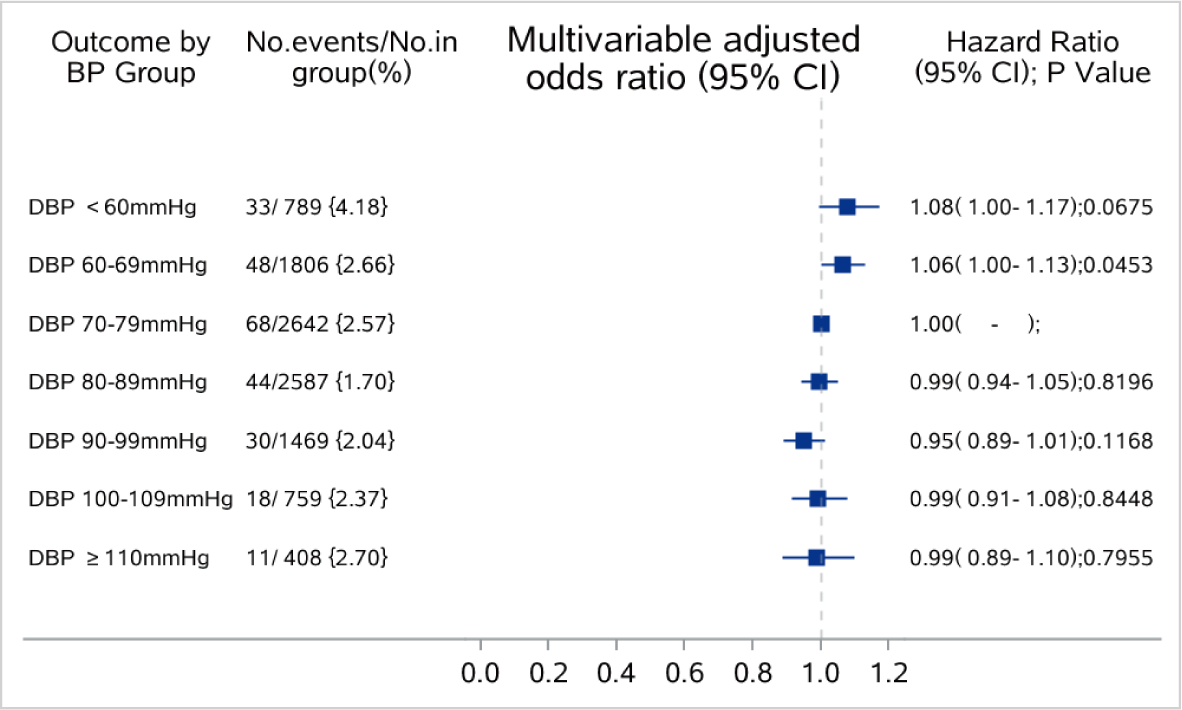

Supplement: Supplementary file 1 [file 2153-8174-26-8-33512-s1.zip › Supplementary Fig. 6/severe bleed-forest plot-DBP.tiff]

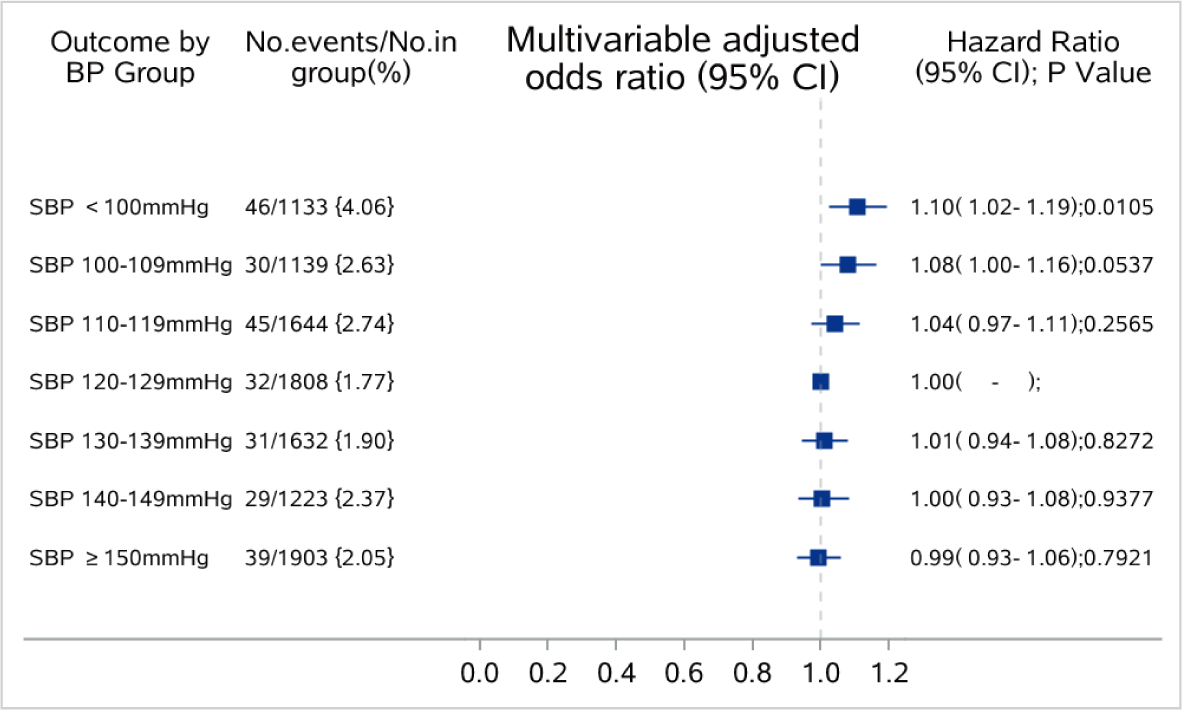

Supplement: Supplementary file 1 [file 2153-8174-26-8-33512-s1.zip › Supplementary Fig. 6/severe bleed-forest plot-SBP.tiff]

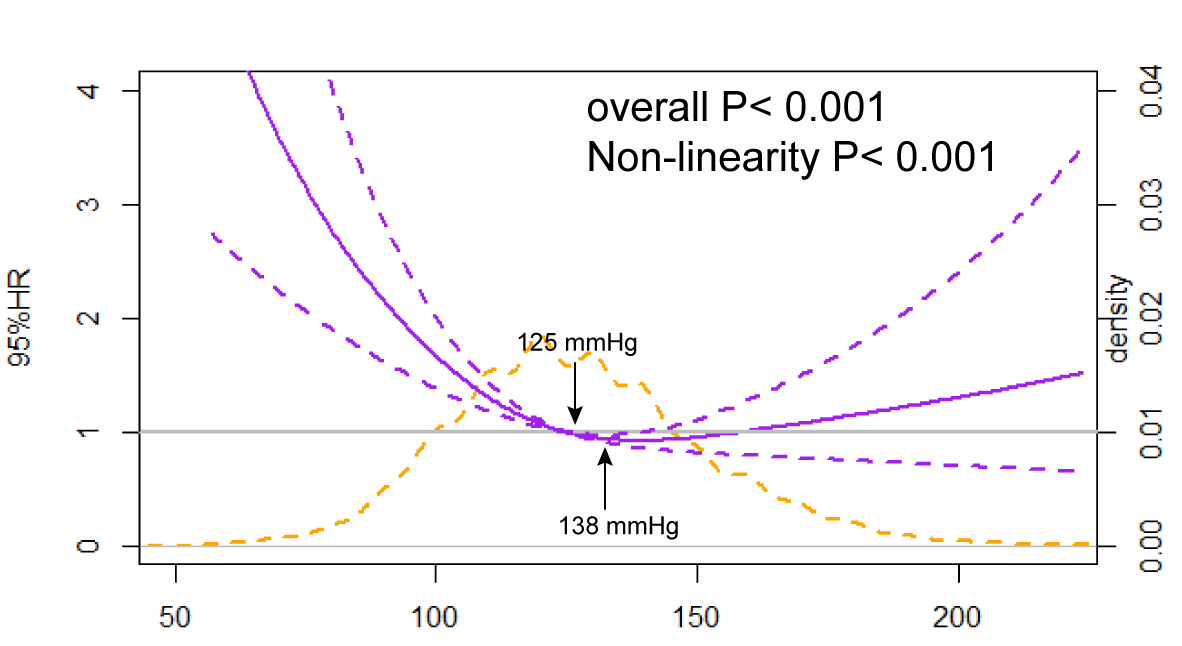

Supplement: Supplementary file 1 [file 2153-8174-26-8-33512-s1.zip › Supplementary Fig. 6/severe bleed-RCS -SBP.tiff]

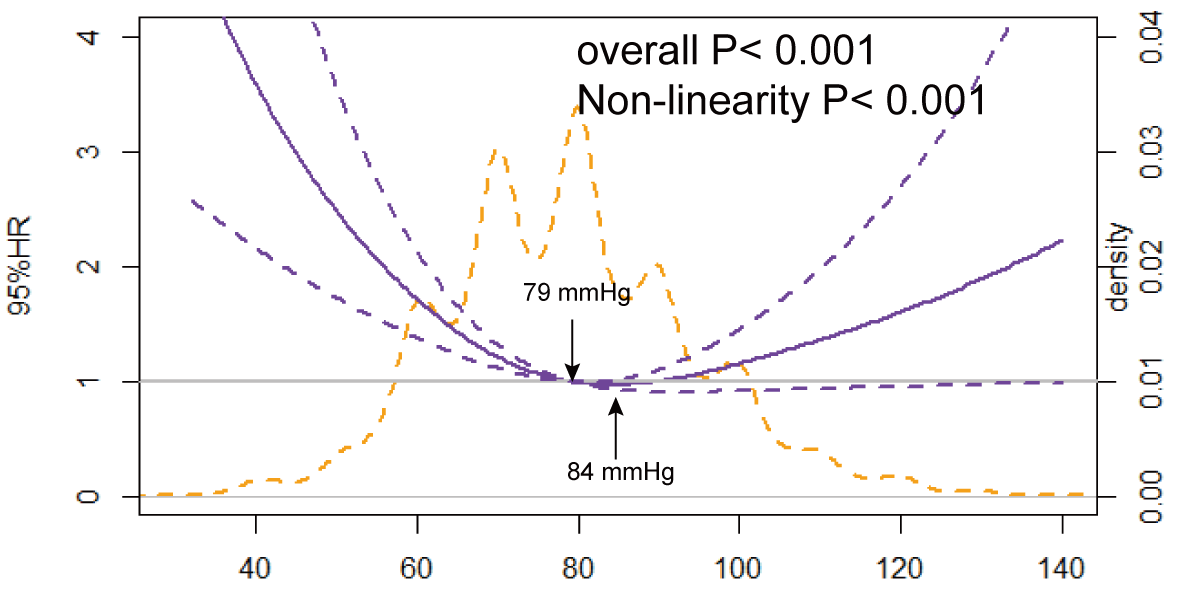

Supplement: Supplementary file 1 [file 2153-8174-26-8-33512-s1.zip › Supplementary Fig. 6/severe bleed-RCS-DBP.tiff]

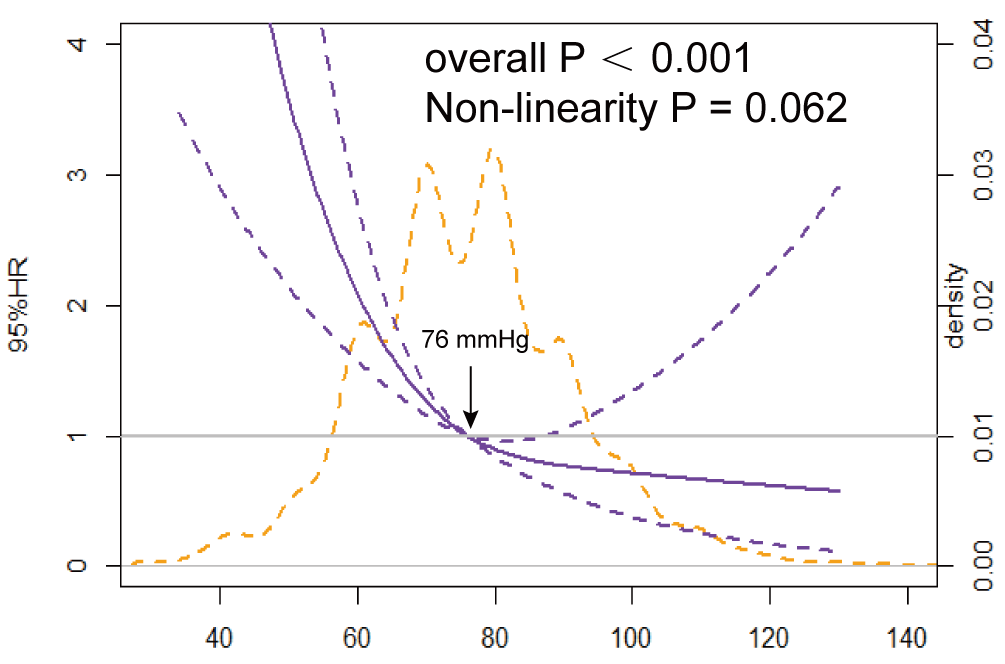

Supplement: Supplementary file 1 [file 2153-8174-26-8-33512-s1.zip › Supplementary Fig. 7/≥65-DBP.tiff]

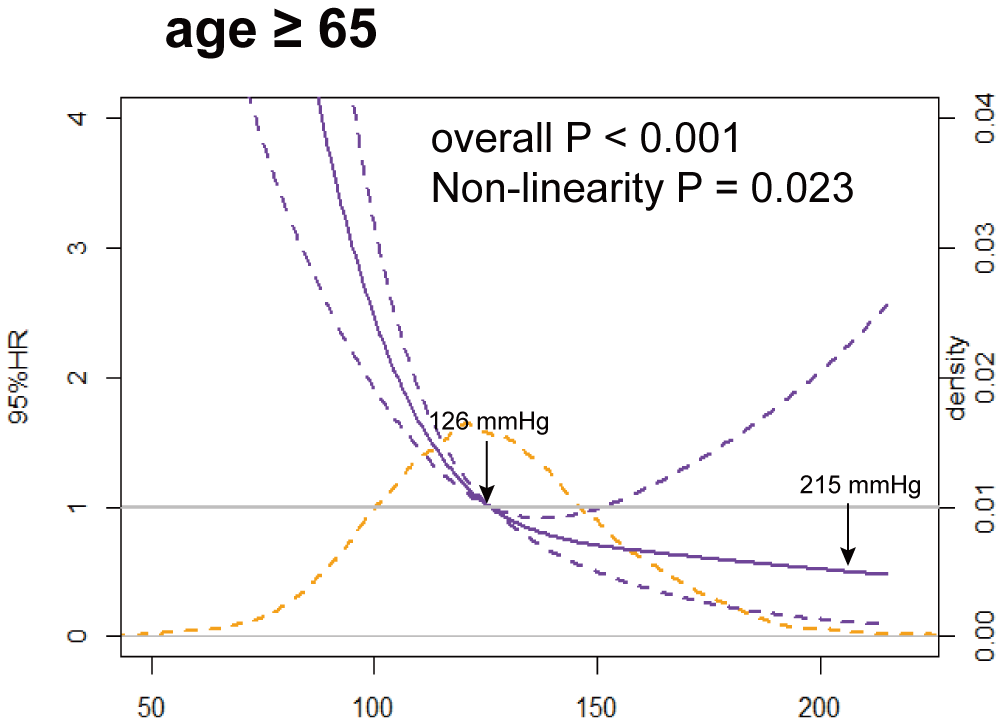

Supplement: Supplementary file 1 [file 2153-8174-26-8-33512-s1.zip › Supplementary Fig. 7/≥65-SBP.tiff]

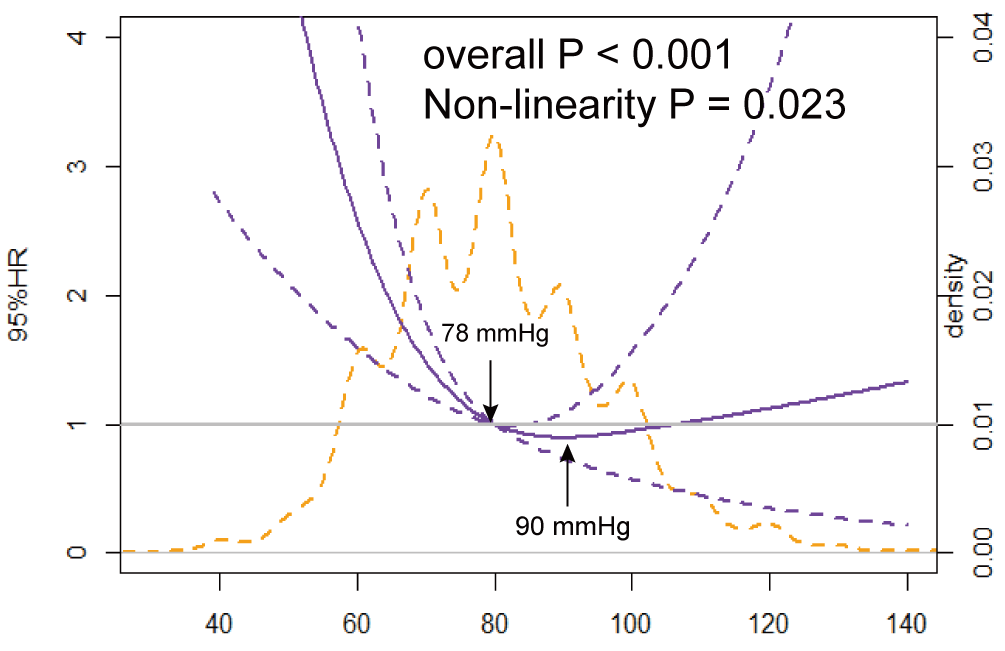

Supplement: Supplementary file 1 [file 2153-8174-26-8-33512-s1.zip › Supplementary Fig. 7/<65-DBP.tiff]

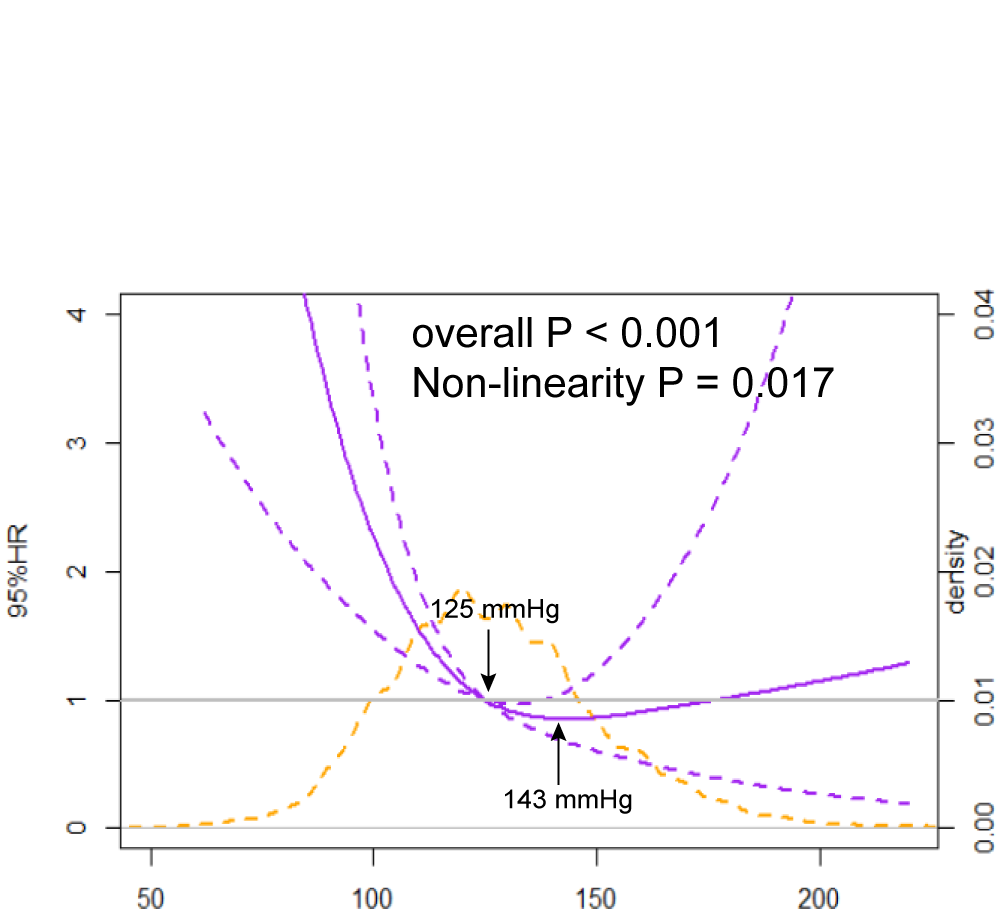

Supplement: Supplementary file 1 [file 2153-8174-26-8-33512-s1.zip › Supplementary Fig. 7/<65-SBP.tiff]

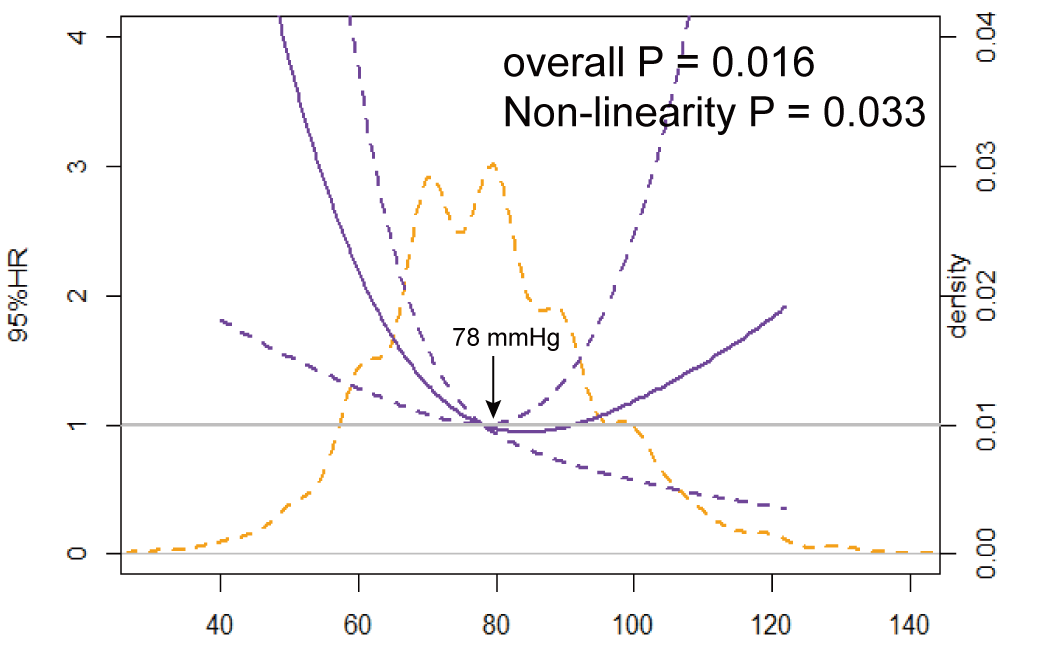

Supplement: Supplementary file 1 [file 2153-8174-26-8-33512-s1.zip › Supplementary Fig. 8/Diabetes-DBP.tiff]

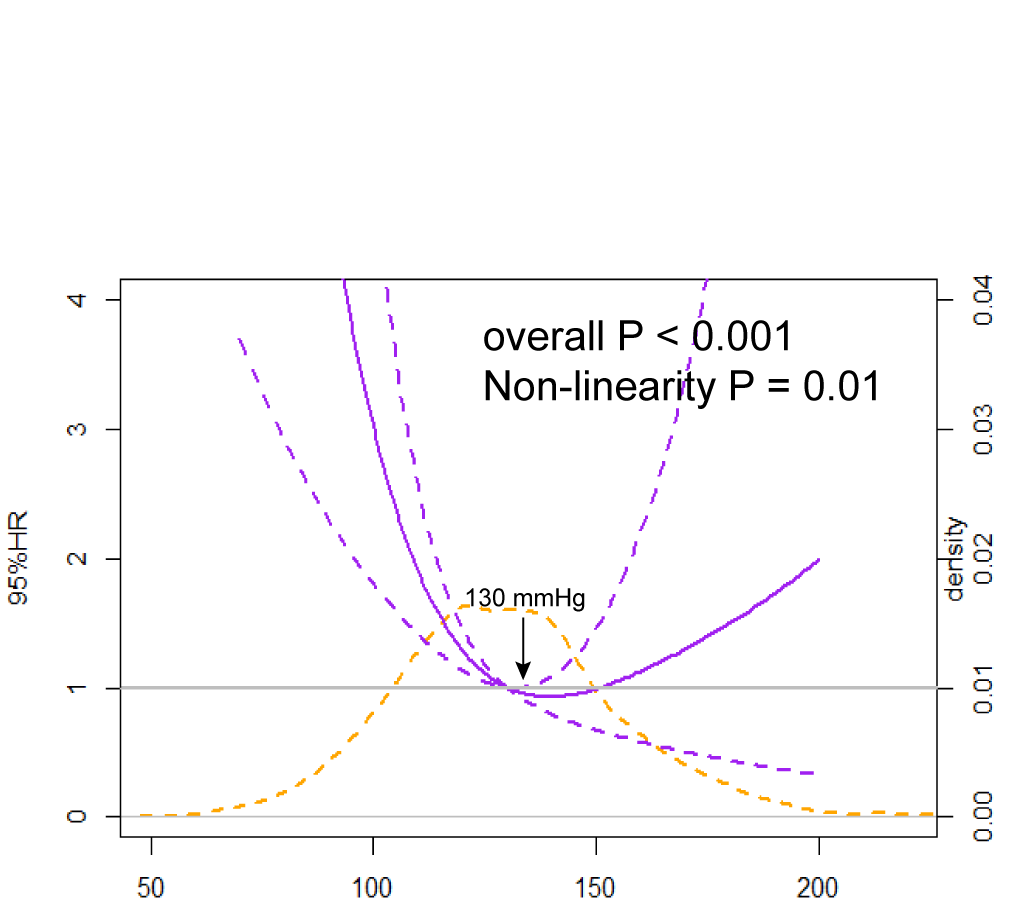

Supplement: Supplementary file 1 [file 2153-8174-26-8-33512-s1.zip › Supplementary Fig. 8/Diabetes-SBP.tiff]

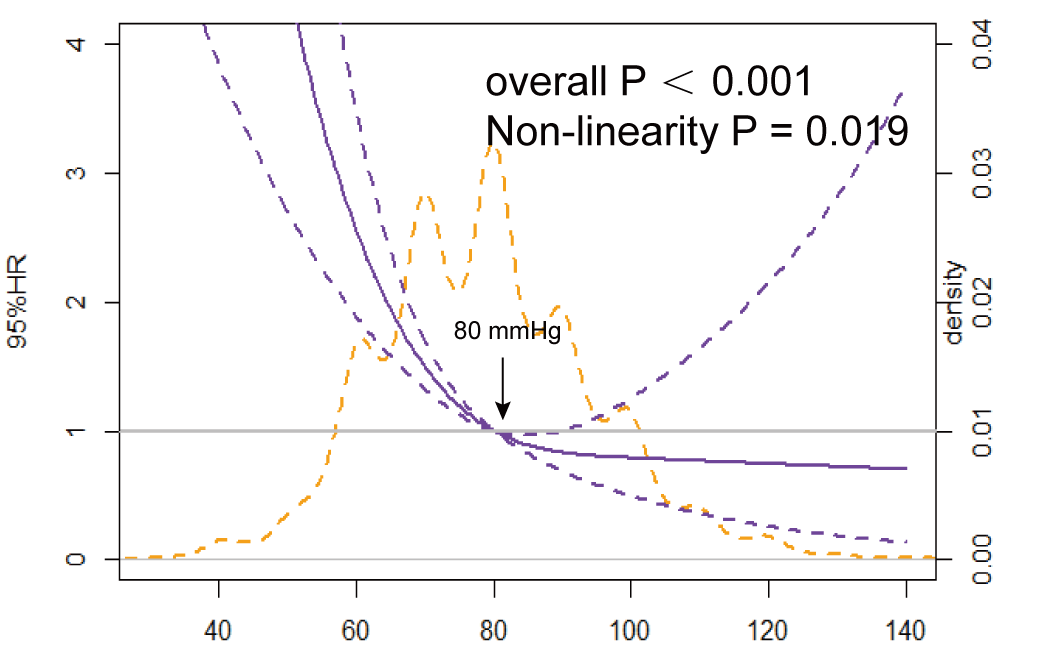

Supplement: Supplementary file 1 [file 2153-8174-26-8-33512-s1.zip › Supplementary Fig. 8/non-Diabetes-DBP.tiff]

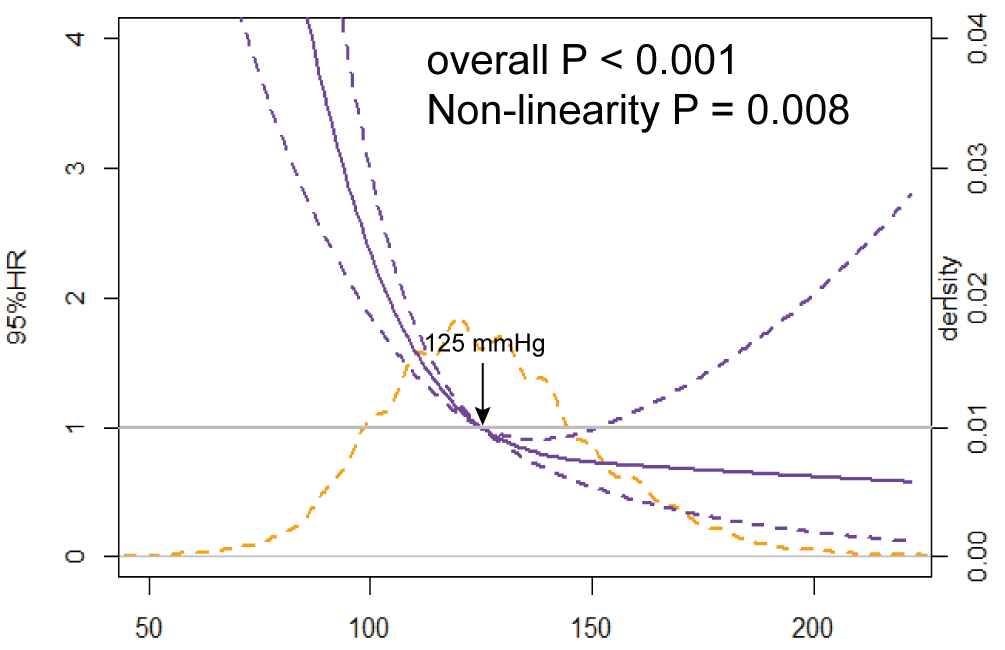

Supplement: Supplementary file 1 [file 2153-8174-26-8-33512-s1.zip › Supplementary Fig. 8/non-Diabetes-SBP.tiff]

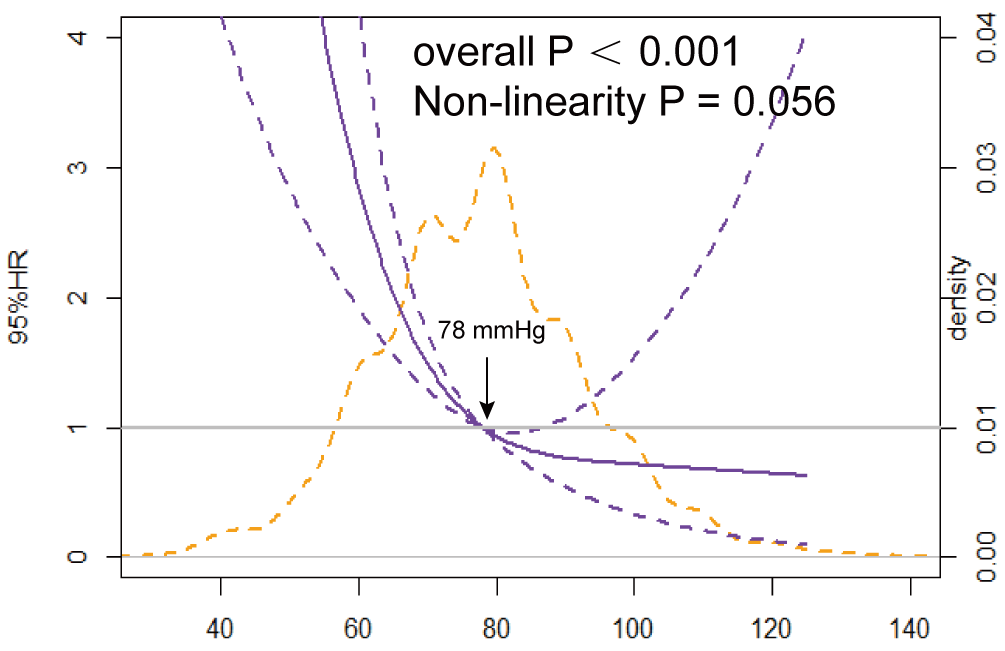

Supplement: Supplementary file 1 [file 2153-8174-26-8-33512-s1.zip › Supplementary Fig. 9/female-DBP.tiff]

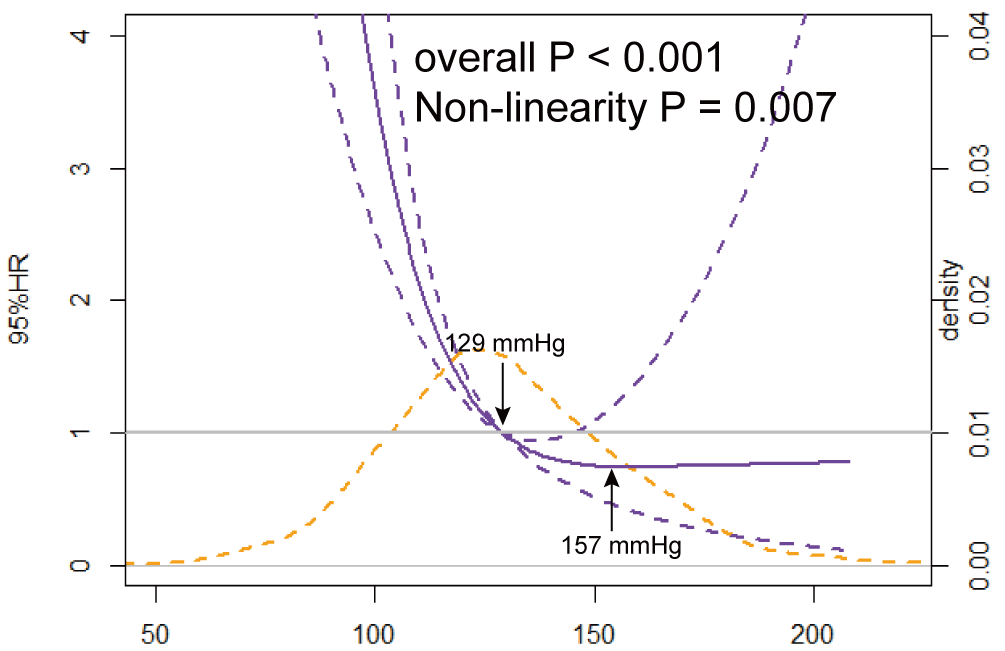

Supplement: Supplementary file 1 [file 2153-8174-26-8-33512-s1.zip › Supplementary Fig. 9/female-SBP.tiff]

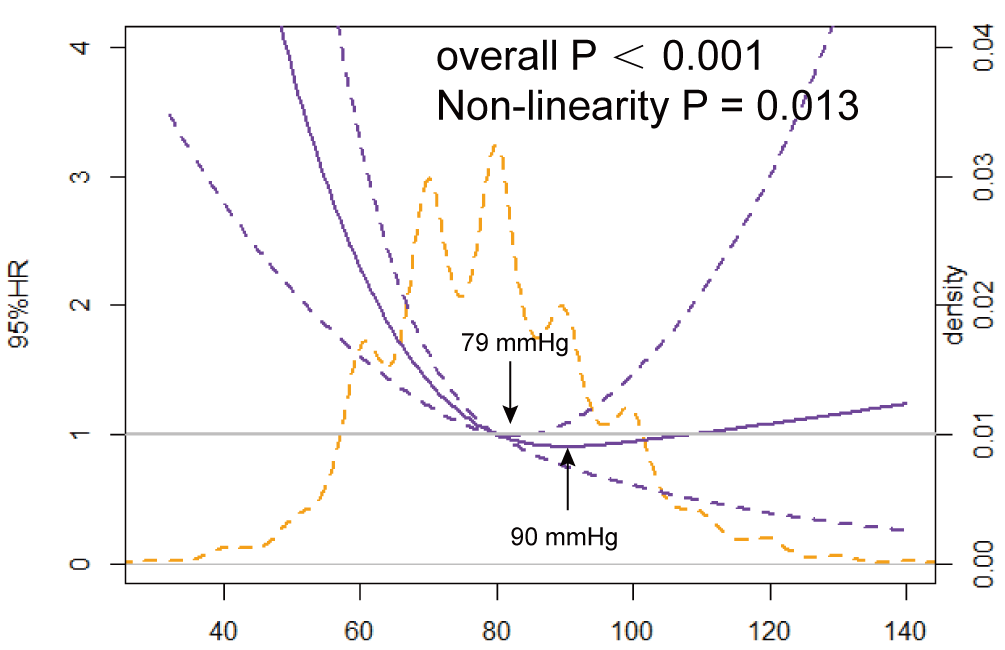

Supplement: Supplementary file 1 [file 2153-8174-26-8-33512-s1.zip › Supplementary Fig. 9/male-DBP.tiff]

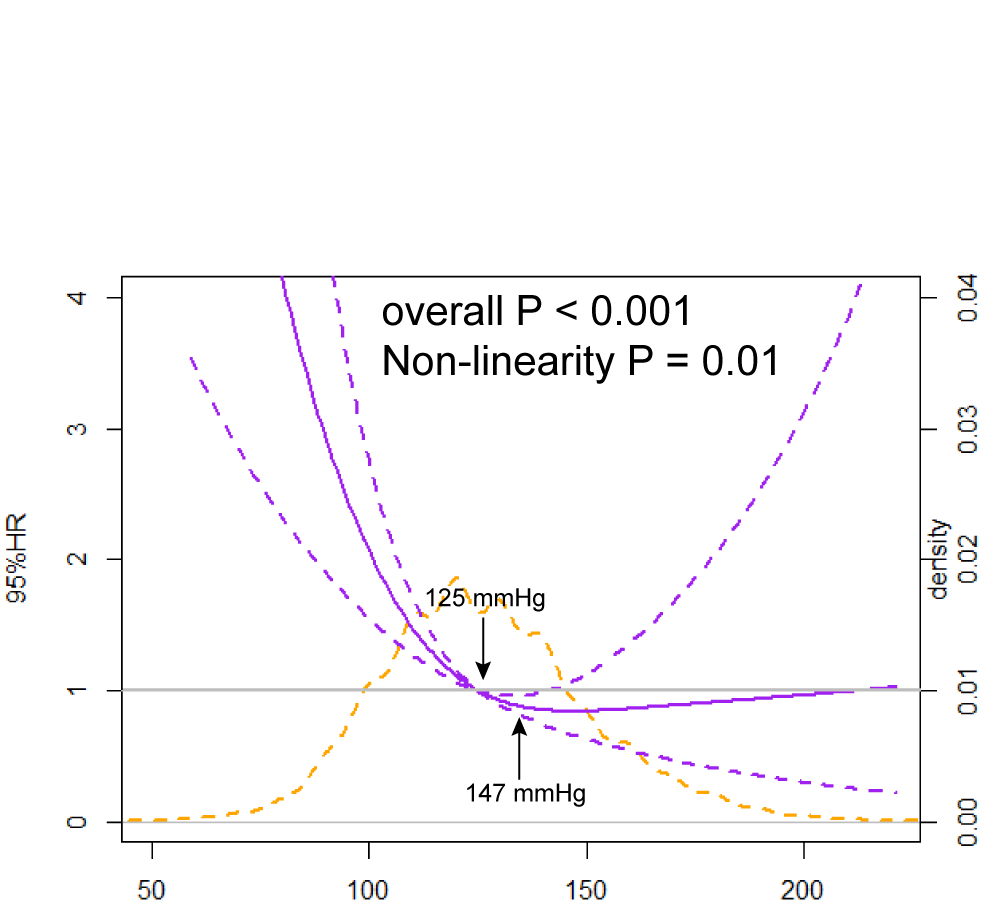

Supplement: Supplementary file 1 [file 2153-8174-26-8-33512-s1.zip › Supplementary Fig. 9/male-SBP.tiff]
